# Human iPSC-derived neural progenitor cells secreting GDNF provide protection in rodent models of ALS and retinal degeneration

**DOI:** 10.1016/j.stemcr.2023.03.016

**Published:** 2023-04-20

**Authors:** Alexander H. Laperle, V. Alexandra Moser, Pablo Avalos, Bin Lu, Amanda Wu, Aaron Fulton, Stephany Ramirez, Veronica J. Garcia, Shaughn Bell, Ritchie Ho, George Lawless, Kristina Roxas, Saba Shahin, Oksana Shelest, Soshana Svendsen, Shaomei Wang, Clive N. Svendsen

**Affiliations:** 1 Cedars-Sinai Board of Governors Regenerative Medicine Institute, Cedars-Sinai Medical Center, Los Angeles, CA, USA

**Keywords:** Stem cell therapy, Neurodegeneration, ALS, Retinitis Pigmentosa, Human iPSC, iPSC, Cell Therapy, CNS, GDNF

## Abstract

Human induced pluripotent stem cells (iPSCs) are a renewable cell source that can be differentiated into neural progenitor cells (iNPCs) and transduced with glial cell line-derived neurotrophic factor (iNPC-GDNFs). The goal of the current study is to characterize iNPC-GDNFs and test their therapeutic potential and safety. Single-nuclei RNA-seq show iNPC-GDNFs express NPC markers. iNPC-GDNFs delivered into the subretinal space of the Royal College of Surgeons rodent model of retinal degeneration preserve photoreceptors and visual function. Additionally, iNPC-GDNF transplants in the spinal cord of SOD1^G93A^ amyotrophic lateral sclerosis (ALS) rats preserve motor neurons. Finally, iNPC-GDNF transplants in the spinal cord of athymic nude rats survive and produce GDNF for 9 months, with no signs of tumor formation or continual cell proliferation. iNPC-GDNFs survive long-term, are safe, and provide neuroprotection in models of both retinal degeneration and ALS, indicating their potential as a combined cell and gene therapy for various neurodegenerative diseases.

## Highlights

• Human iNPC-GDNFs differentiate into astrocytes both *in vitro* and *in vivo*
• iNPC-GDNFs can protect cells and function in the diseased rat retina and spinal cord
• iNPC-GDNFs show long-term survival and GDNF production in the nude rat spinal cord
• iNPC-GDNFs provide a safe and expandable combined cell and gene therapy

## Graphical abstract

**Figure:**
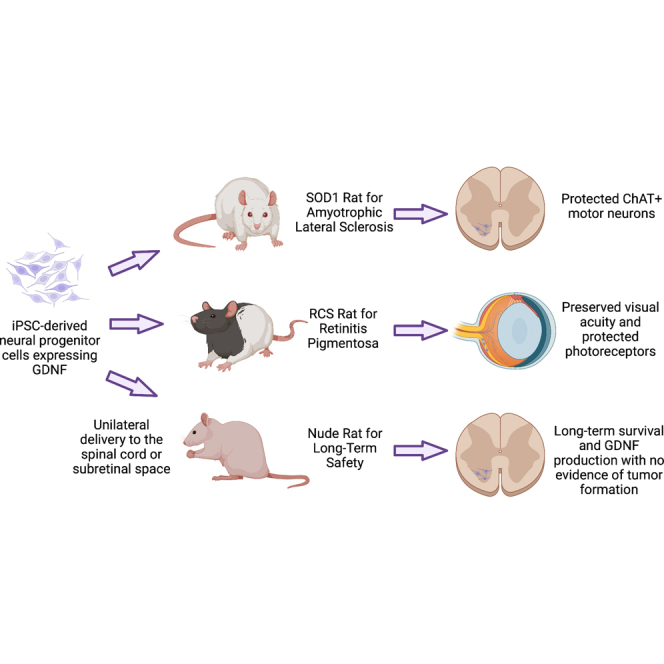

## Teaser

Direct replacement of lost cells in neurodegenerative conditions remains challenging. Alternatively, diseased cells can be protected by delivering astrocytes and growth factors. Laperle et al. demonstrate that iPSC-derived neural progenitor cells can be engineered to stably produce GDNF (iNPC-GDNF), which survive in the central nervous system, are safe, can slow cell death in multiple disease models, and protect function.

## Introduction

Several neurodegenerative diseases, including Parkinson’s and Alzheimer’s, and amyotrophic lateral sclerosis (ALS), involve unique genetic and environmental risk factors, while others such as retinitis pigmentosa (RP) involve various genetic mutations. Replacing the damaged cell population is one treatment approach; however, transplanted cells do not readily form new long-distance synaptic connections with their targets or integrate with secondary retinal neurons in the adult environment. Therefore, protection of remaining host cells is a more practical approach and suggests the need for a protective intervention that is beneficial across various neurodegenerative diseases.

ALS involves progressive motor neuron death in the spinal cord and motor cortex, leading to paralysis and death, typically within 2–5 years of diagnosis (Hulisz, 2018). There are currently no effective treatments. As glial cells are compromised in ALS, one neuroprotective strategy is to provide new glial support cells (Bruijn et al., 1997; Lepore et al., 2008). These can be derived from the human fetal forebrain and then sorted to isolate glial-restricted progenitors (GRPs) (Lepore et al., 2011; Sandrock et al., 2010). Alternatively, we have shown that human cortical fetal-derived neural progenitor cells (fNPCs) can be expanded in culture as free-floating spheres (Svendsen et al., 1998) and can differentiate into astrocytes *in vitro* and *in vivo* (Das et al., 2016; Gowing et al., 2014; Svendsen et al., 1997). While GRP and fNPC transplants survive in the spinal cord of the well-characterized SOD1^G93A^ (hereto, SOD1) transgenic rodent model of ALS, neither benefit motor neuron survival nor functional measures. This suggests that an additional strategy may be required for neuronal protection. As glial cell line-derived neurotrophic factor (GDNF) has protective effects on dopamine and motor neurons (Henderson et al., 1994; Lin et al., 1993; Zurn et al., 1994), we have genetically engineered fNPCs to stably secrete GDNF (fNPC-GDNF) (Capowski et al., 2007). Unlike fNPCs alone, this combined cell and growth factor *ex vivo* therapy could protect spinal motor neurons in the SOD1 ALS rat, as well as dopamine neurons in a Parkinsonian rat model (Behrstock et al., 2006; Klein et al., 2005; Suzuki et al., 2007). In addition, delivery of fNPCs releasing GDNF to the ALS rat motor cortex protected motor neurons, slowed disease progression, and extended lifespan (Thomsen et al., 2018). RP involves progressive loss of photoreceptors, ultimately leading to blindness. Using the well-established Royal College of Surgeons (RCS) rat model of retinal degeneration, we have shown that fNPCs can protect vision and photoreceptors and that GDNF-secreting fNPCs provide enhanced protection (Gamm et al., 2007).

We previously expanded a single fetal cortical sample under current Good Manufacturing Practice (cGMP) to derive a neural progenitor cell line, termed CNS10-NPC, which was lentivirally transduced to stably produce GDNF and banked as a clinical product (termed CNS10-NPC-GDNF) (Shelley et al., 2014). This product was used for a first-in-man cell and gene therapy for ALS with delivery to the lumbar spinal cord in a recently completed phase I/IIa safety trial (clinical trials.gov: NCT02943850). The primary endpoint of safety at 1 year was met, with no negative effect of the transplant on motor function, and grafts survived and produced GDNF for up to 42 months post-transplantation (Baloh et al., 2022). CNS10-NPC-GDNF is now being delivered to the motor cortex of ALS patients in a phase I/IIa clinical trial (NCT05306457). Finally, a current phase I/IIa clinical trial is transplanting CNS10-NPC into the subretinal space of RP patients (NCT04284293).

Fetal-derived NPCs and their derivatives are a promising treatment for Parkinson’s disease, ALS, and RP, as well as other neurological conditions such as Huntington’s disease, stroke, and dementia (Andres et al., 2011; Goldberg et al., 2017; McBride et al., 2004). However, this product has some limitations, including low fetal tissue availability that can hinder large scale-up, thus restricting phase III trials and commercialization. Our lab and others have shown that induced pluripotent stem cells (iPSCs) provide a renewable, scalable, and safe cell source for deriving potential cell products (Rosati et al., 2018; Sareen et al., 2014; Tsai et al., 2015). Peripheral blood mononuclear cells (PBMCs) from an individual’s blood sample can be reprogrammed into iPSCs, which proliferate in culture, differentiate into multiple tissue types, and can be cryopreserved and thawed (Barrett et al., 2014). We have demonstrated that human iPSCs cultured in specified media could generate neural-specific cultures that were propagated as spheres (termed EZ spheres), which could engraft and differentiate into astrocytes in the rodent spinal cord and retina (Ebert et al., 2013; Sareen et al., 2014; Tsai et al., 2015).

Here, we developed and tested an iPSC-based therapy as an alternative to fetal-derived products. We generated iPSC-derived NPCs (termed iNPCs), which were transduced to express GDNF (iNPC-GDNF), characterized, and compared with fNPC-GDNFs. iNPC-GDNFs were transplanted into the subretinal space of RCS rats where they protected photoreceptors and vision, the spinal cord of SOD1 rats where they protected motor neurons, and the spinal cord of nude rats where they showed long-term survival and safety. Based on safety and efficacy, iNPC-GDNFs can be pursued as a promising combined cell and gene therapy for multiple neurodegenerative diseases.

## Results

### iNPC-GDNFs and derived astrocytes resemble fNPC-GDNFs and derived astrocytes

**Figure 1:**
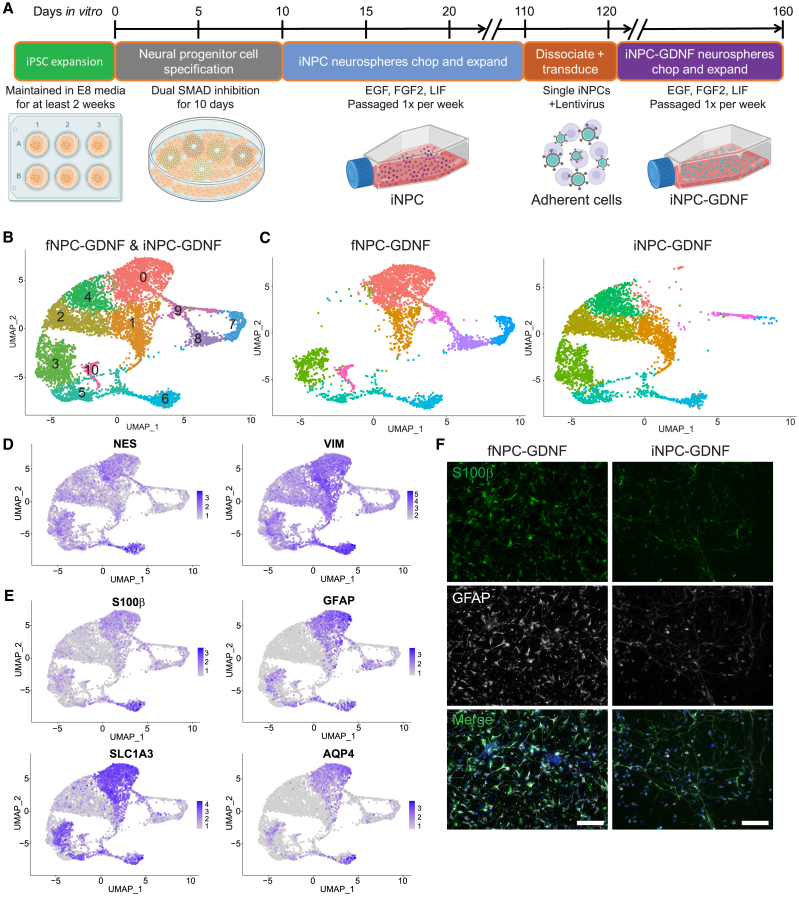
iNPC-GDNF and fNPC-GDNF profiles (A) Protocol to generate and expand iNPC-GDNFs. (B) Unbiased UMAP clustering of cells from fNPC-GDNF and iNPC-GDNF neurospheres split into 11 defined clusters. (C) Clustering split by cell type shows that iNPC-GDNFs clustered similarly to fNPC-GDNFs in many but not all cases. (D and E) Feature plots show expression of (D) cortical neural progenitor markers and (E) astrocyte markers. (F) Immunocytochemistry on fNPC-GDNFs and iNPC-GDNFs differentiated for 7 days in culture shows production of S100β and GFAP, with DAPI (blue). Scale bar represents 75 μm. See also Table S1 and Figure S1.

NPCs derived from a single human fetal cortex were maintained as free-floating spheres (termed neurospheres), expanded by mechanical passage using a tissue chopper (Shelley et al., 2014; Svendsen et al., 1998), transduced with lentivirus to stably express GDNF (Capowski et al., 2007; Shelley et al., 2014), then propagated and banked under cGMP as the CNS10-NPC-GDNF clinical cell lot, and fNPC-GDNFs were banked as a research-grade cell lot used in this study. To produce iPSC-derived NPCs similar to these fNPCs, a new and substantially shorter protocol was developed. An iPSC line was generated by nucleofecting healthy PBMCs with nonintegrating oriP/EBNA1 plasmids, which allowed for episomal expression of reprogramming factors (Barrett et al., 2014). Using dual SMAD inhibition (Chambers et al., 2009), a monolayer of neuroepithelial progenitors was efficiently generated, which were then collected and transitioned into neurospheres in the presence of epidermal growth factor (EGF), fibroblast growth factor 2 (FGF2), and leukemia inhibitory factor (LIF) (Figure 1A). iNPC neurospheres were expanded by mechanical passage using a tissue chopper or mesh chopper. After passage 16, iNPCs were dissociated to single cells and lentivirally infected to stably express GDNF. Transduced cells reformed as iNPC-GDNF neurospheres that were used in the current studies.

To demonstrate consistency in the production process, two independent batches of iNPC-GDNFs were produced. G-band karyotyping revealed that one batch remained karyotypically normal, while the other batch acquired a karyotypic anomaly not present in the originating iPSC line with nearly 100% trisomy of chromosome 12, termed iNPC-GDNF-T12 (Figure S1A). To avoid complications that could arise from karayotypic abnormality, subsequent studies were performed with the karyotypically normal iNPC-GDNF batch, which remained normal up to 19 passages. To identify any potential remaining pluripotent cells in the differentiated batches, pluripotency genes *POU5F1 (OCT4), NANOG, SOX2,* and *KLF4* were plotted from single-nuclei RNA sequencing (snRNA-seq) data (Figure S1B). While individual cells expressed some of these markers at low levels, no cell expressed all four or any combination of *OCT4, NANOG,* and *KLF4* (Figure S1C). As expected, *SOX2* was expressed throughout most cells (Figure S1B), as it is also a NPC marker (Zhang and Cui, 2014).

snRNA-seq was performed to assess the similarity between iNPC-GDNF and fNPC-GDNF neurosphere cultures. Following batch normalization, UMAP (uniform manifold approximation and projection) was applied to identify clusters of cell types in an unbiased manner, which identified 11 separate clusters (Figure 1B, Table S1). When split by sample source, fNPC-GDNFs were found in all clusters except for 2 and 4, which were almost exclusively iNPC-GDNFs, while clusters 8 and 10 were entirely fNPC-GDNFs (Figure 1C, Table S1). Importantly, the overlap of a population of iNPC-GDNF clusters in most fNPC-GDNF clusters demonstrates the remarkable similarity in some cell populations. Indeed, markers of cortical NPCs were highly expressed throughout all cells in this analysis (Figure 1D). Exclusively iNPC-GDNF clusters tended to express markers for the immature progenitors (*VIM*, *SOX2*, *NESTIN*). Interestingly, clustering was often driven by cell cycle phase (Figure S1D), again indicating strong similarities between cell types, as this distinction does not commonly appear in more diverse datasets. Cluster 0 cells showed high expression of astrocyte-related genes (Figure 1E). Given that this cluster had a low contribution in iNPC-GDNF neurospheres (Table S1), we wanted to assess their differentiation potential. iNPC-GDNFs and fNPC-GDNFs were differentiated as monolayer cultures for 7 days, with subsequent immunocytochemistry showing that both produced the astrocyte markers glial fibrillary acidic protein (GFAP) and S100β (Figure 1F). Flow cytometry further demonstrated that 80% of differentiated iNPC-GDNFs acquired GFAP production (Figure S1E). Collectively, results demonstrate that while some differences exist in fetal- and iPSC-derived NPCs, there also are substantial similarities.

### iNPC-GDNFs preserve vision and photoreceptors in the RCS rat model

**Figure 2:**
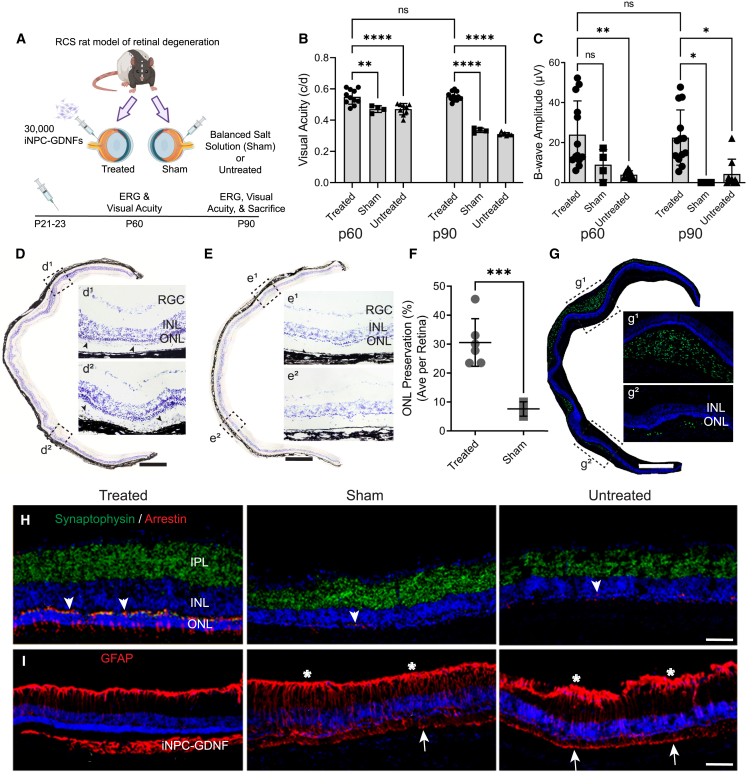
iNPC-GDNFs are neuroprotective in the RCS rat model of retinal degeneration (A) Schematic of experiment RCS rats (n = 13) that received subretinal injection of iNPC-GDNFs at postnatal day (P) 21–23. The fellow eye received either balanced salt solution injection (sham, n = 4) or no treatment (n = 9). (B and C) Visual function was evaluated by (B) OKR and (C) ERG, which showed significant preservation in cell-treated compared with control animals (^∗∗∗∗^p < 0.0001, ^∗∗^p < 0.01, ^∗^p < 0.05, one-way ANOVA with Tukey’s HSD, ns: not significant). (D and E) Cresyl violet-stained retinal images show photoreceptor preservation in (D) iNPC-GDNF-treated retina with potential grafted cells (arrowheads), compared with (E) sham. d^1^, d^2^, e^1^, and e^2^ are high-power images from areas in boxed outlines. (F) Percent of retina with at least two layers of photoreceptors in iNPC-GDNF-treated vs. sham (^∗∗∗^p < 0.001 via unpaired t test with Welch’s correction). (G) Staining with human nuclear marker MAB1281 shows extensive distribution of grafted iNPC-GDNFs; g^1^ and g^2^ are high-power images from areas in boxed outlines. (H) Synaptophysin and cone arrestin staining show cones with segments and pedicles (arrowheads) were preserved in cell-treated retina compared with controls. (I) GFAP staining shows reactive Müller glia in controls and cell-treated retina, GFAP labels iNPC-GDNFs, and host Müller glia (stars, arrows point to Müller glial end feet). Scale bars represent (D, E, and G) 500 μm and (H and I) 75 μm. INL, inner nuclear layer; IPL, inner plexiform layer; ONL, outer nuclear layer. d^1^, e^1^, and g^1^ indicate regions near injection site, d^2^, e^2^, and g^2^ indicate regions distal from injection site. See also Figure S2.

Fetal- and iPSC-derived NPCs can protect vision following a single subretinal injection into the RCS rat (Gamm et al., 2007; Tsai et al., 2015; Wang et al., 2008). Importantly, GDNF-secreting fNPCs provide enhanced vision protection, and GDNF delivery can preserve photoreceptors (Gamm et al., 2007; García-Caballero et al., 2018). To evaluate the efficacy of iNPC-GDNFs, RCS rats at postnatal day (P) 21–23 received a single unilateral subretinal injection of cells, and the fellow eye served as the control, with balanced salt solution (sham) injection or no treatment (Figure 2A).

Visual acuity measured by the optokinetic response (OKR) showed that sham-treated and untreated eyes had a significant decrease in visual acuity from P60 to P90 (Figure 2B). However, there was almost complete rescue with iNPC-GDNF treatment, with visual acuity over time remaining around 0.55 cycle/degree (Figure 2B), comparable to wild-type rats (McGill et al., 2007). Electroretinography (ERG), measuring the average response of the whole retina to light stimulation, revealed that iNPC-GDNF-treated eyes had significantly higher b-wave amplitude compared with controls at P60 and P90 (Figure 2C). ERG does show visual impairment compared with a wild-type rat, as ERG was compromised already when treatment started. But, critically, iNPC-GDNF treatment provided sustained protection of both ERG and OKR over the study duration.

To assess protection of retinal morphology at P90, the length of preserved photoreceptors was measured against the whole length of cresyl violet-stained retinal sections, which showed that iNPC-GDNF treatment preserved three to six photoreceptor layers, compared with most controls with only one layer remaining (Figures 2D and 2E). Quantifying the outer nuclear layer (ONL) showed that iNPC-GDNFs protected an average of 30.59% ± 3.6% of the retina, indicating roughly one-third of the retinal sections retained at least two layers of photoreceptors (Figure 2F). Further, staining with the human-specific nuclear antibody MAB1281 confirmed iNPC-GDNFs had robust survival and extensive migration in the subretinal space (Figure 2G) and, notably, demonstrated multiple layers of photoreceptors associated with donor cells at the injection site (Figure 2G^1^) and with migrating donor cells (Figure 2G^2^). iNPC-GDNF treatment also preserved cone photoreceptors, revealed by immunofluorescence with a cone arrestin antibody that showed inner and outer segments and dense cone pedicles (Figure 2H). In contrast, controls had only weak staining for cones, with no visible segments and cone pedicles. Retinal connections, based on wide and disperse synaptophysin staining, were evident in cell-treated retinas, compared with narrow staining in controls (Figure 2H). Strong GFAP staining in control retinas indicates a reactive Müller glia response (Figure 2I). This was substantially reduced in the treated retina and mainly located in the optic nerve fiber layer, suggesting that cell treatment reduced Müller glia activation. The pan-GFAP antibody also stained grafted iNPC-GDNFs, indicating differentiation into astrocytes.

Immunofluorescence with human-specific antibodies for Nestin and astrocytes (SC123) was used to characterize grafted iNPC-GDNFs, which showed that cells remained as neural progenitors or differentiated into astrocytes (Figures S2A and S2B). Consistent with our prior study (Gamm et al., 2007), a few grafted cells migrated into the inner retina, but most formed a layer of cells or lump within the subretinal space. While Ki67 staining indicates proliferating host cells in the degenerative environment, only a few Ki67-positive human cells demonstrated the limited proliferation of transplanted iNPC-GDNFs (Figure S2C).

### iNPC-GDNFs are neuroprotective in SOD1 ALS rat

**Figure 3:**
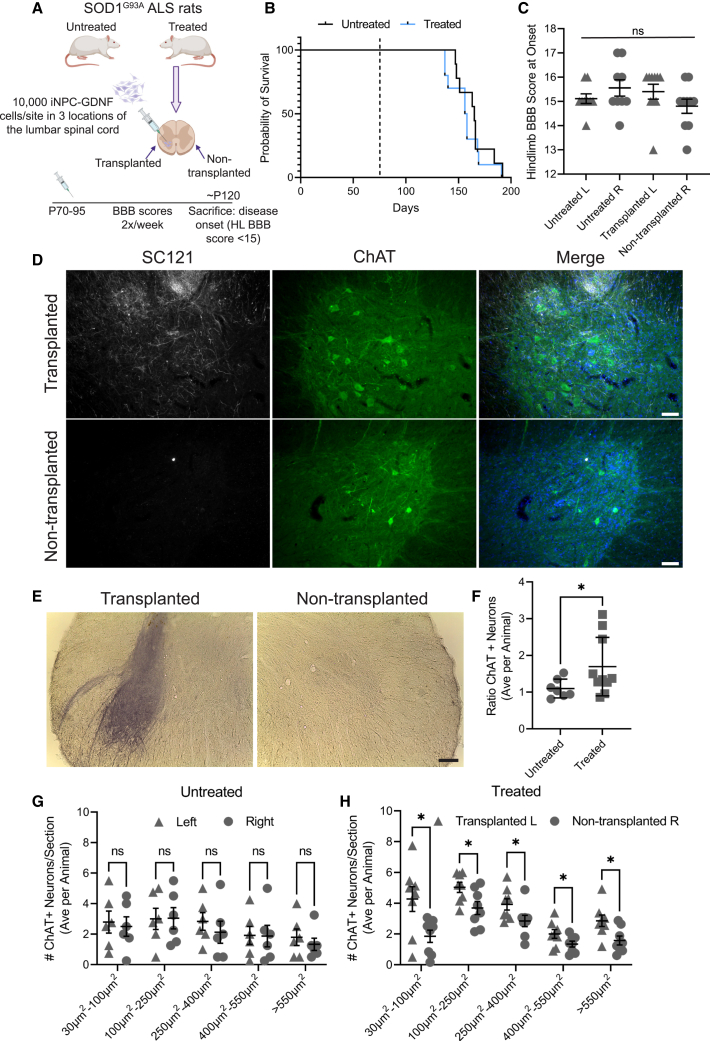
iNPC-GDNFs are neuroprotective in the SOD1 ALS rat (A) Schematic of experiment. 10 male SOD1 rats at 70–95 days were unilaterally transplanted (left, L) with 10,000 iNPC-GDNFs in three sites 2 mm apart in the lumbar spinal cord and compared with 9 untreated animals. (B) Kaplan-Meier shows probability of onset times of treated vs. untreated animals are not significantly different. (C) Hindlimb BBB scores at disease onset are not significantly different. (D) Immunohistochemistry shows SC121+ human grafts surrounding host ChAT+ motor neurons, with DAPI (blue). (E) GDNF staining of transplanted compared with non-transplanted spinal cord. (F) Ratio of ChAT+ motor neurons averaged per animal (n = 7 untreated and n = 10 treated animals). (G and H) ChAT+ neuron size in (G) untreated animals (n = 6, average of four sections per animal) and (H) treated animals (n = 8, average of at least eight sections per animal); ^∗^p < 0.05 via unpaired t test with Welch’s correction (F) or multiple paired t tests corrected with the Holm-Šídák method (G and H); ns: not significant. Scale bars represent (D) 75 μm and (E) 250 μm. See also Figure S3.

To evaluate whether iNPC-GDNFs are protective in SOD1 ALS rats, cells were injected unilaterally into the lumbar spinal cord, with untreated SOD1 rats serving as controls (Figure 3A). Treated and untreated groups had similar body weights over the study course (Figure S3A). Basso, Beattie, and Bresnahan (BBB) locomotor scores were collected weekly, starting 1 week before transplantation, to assess hindlimb function and disease onset. There was no significant difference in BBB scores for transplanted/non-transplanted hindlimbs in treated and untreated rats, indicating no delay in disease onset (Figures 3B and 3C).

To assess iNPC-GDNF survival and host motor neurons, rats were euthanized at disease onset, and tissue was analyzed by immunohistochemistry. Grafted cells stained positive for the human-specific cytoplasmic marker (SC121) with some cells co-staining for the neural progenitor marker Nestin (Figure S3B). Minimal co-staining of SC121 with the neuronal marker Neun (Figure S3C) coupled with extensive co-staining for GFAP (Figure S3D) indicate a primarily glial identity of the grafted cells. SC121 staining confirmed iNPC-GDNFs in the treated spinal cord, but not the non-treated side, with engraftment around choline-o-acetyl transferase (ChAT)-positive host motor neurons (Figure 3D). Robust GDNF production was seen in the iNPC-GDNF-treated spinal cord but not in the non-transplanted side (Figure 3E). While all transplanted animals showed surviving grafts, the location and extent varied (Figures S3E and S3F), and it was observed that better graft targets correlated with greater motor neuron counts. To assess whether iNPC-GDNFs protected host motor neurons, spinal cord sections containing SC121+ grafts were quantified for ChAT+ motor neuron numbers and size. ImageJ analysis revealed a significantly elevated ratio of total number of motor neurons (transplanted/non-transplanted) in treated compared with untreated animals (Figure 3F). We have previously shown that fNPC-GDNF specifically protect large motor neurons (<600 μM) in the ALS rat spinal cord and motor cortex (Suzuki et al., 2007; Thomsen et al., 2018). Here, the number of ChAT+ motor neuron across all size bins was significantly increased between transplanted and non-transplanted sides in treated animals, with no difference across size bins between the left and right spinal cord in untreated animals (Figures 3G and 3H). This indicates that iNPC-GDNFs provide neuroprotection of motor neurons in the ALS rat spinal cord.

### iNPC-GDNFs show long-term survival and safety in the nude rat spinal cord

**Figure 4:**
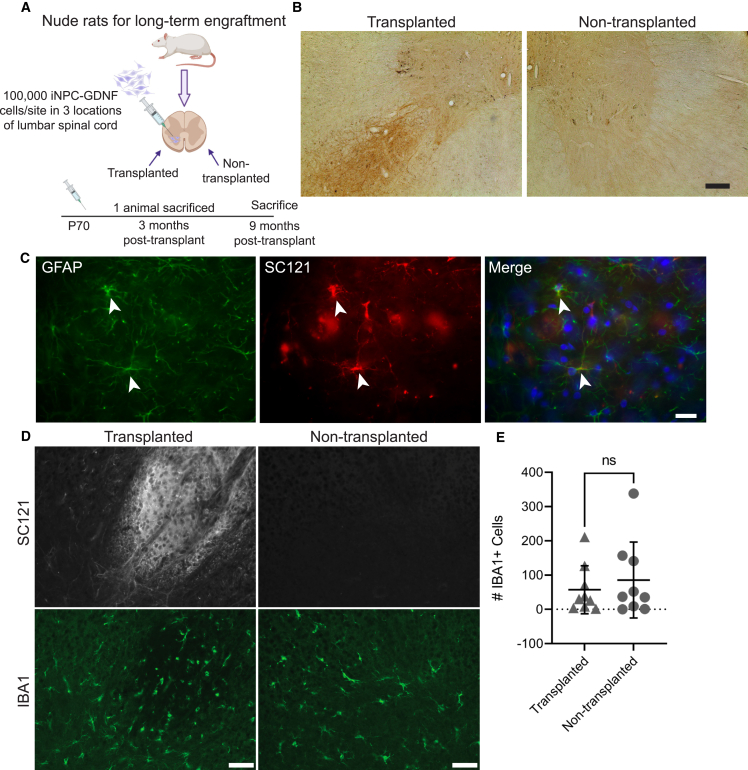
iNPC-GDNF spinal cord long-term grafts (A) Schematic of experiment. Rats (n = 10) were unilaterally transplanted with 100,000 iNPC-GDNFs in three sites 2 mm apart in the lumbar spinal cord, with euthanasia at 3 months (n = 1) and 9 months (n = 9) post-transplantation. (B and C) Grafts at 9 months were stained for (B) GDNF and (C) pan-GFAP and SC121 with DAPI (blue). (D and E) SC121 and IBA1 staining (D) and quantification of IBA1+ cells with a paired t test (E) (average per animal, minimum of four sections analyzed per animal). ns: not significant. Scale bars represent (B) 75 μm, (C) 20 μm, and (D) 50 μm. See also Figure S4.

To examine iNPC-GDNF long-term survival and safety, cells were transplanted unilaterally into the lumbar spinal cord of athymic nude rats (Figure 4A). These rats have a spontaneous mutation in the *Foxn1* gene and thus lack T cells, rendering them immunodeficient to enable xenograft survival without immunosuppression. iNPC-GDNF transplants survived well in both gray matter and white matter spinal cord regions (Figures S4A and S4B) and produced GDNF (Figure 4B) for up to 9 months. Histopathological examination of hematoxylin and eosin staining showed no structural abnormalities indicative of cancerous growth in any section across all animals. Ki67-postive cells were observed at 3 months but not 9 months post-transplantation, indicating reduced proliferation over time (Figures S4C and S4D). At 9 months post-transplantation, some iNPC-GDNFs retained Nestin expression (Figure S4E) with relatively little overlap of Neun (Figure S4F). Strong staining from an antibody that recognizes human and rat GFAP (pan-GFAP) and from a human-specific GFAP antibody (SC123) demonstrated that cells differentiated primarily into astrocytes (Figure S4G), and higher magnification shows clear astrocyte morphology for iNPC-GDNFs co-labeled with SC121 and GFAP (Figure 4C).

To examine effects of long-term iNPC-GDNF transplants on spinal cord inflammation, immunohistochemistry was performed for human cells and the microglial marker IBA-1 (Figure 4D). While microglia number varied across animals, there was no significant increase in microglia surrounding the SC121+ graft compared with the contralateral spinal cord (Figure 4E), indicating that iNPC-GDNFs did not enhance long-term inflammation. Collectively, results demonstrate that long-term iNPC-GDNF transplants can survive, differentiate into glia, and are safe with no evidence of tumor growth.

## Discussion

Neurodegeneration encompasses many different diseases and cell types with diverse genetic and environmental causes that elude pathway-targeted therapies. Instead, NPCs that engraft and differentiate into supportive glia, and which can be genetically engineered to produce a growth factor, are broadly applicable to many diseases. A focal delivery approach is critical as GDNF cannot cross the blood-brain barrier, and systemic delivery has been associated with adverse effects (Thomsen et al., 2017). A combined cell and gene therapy approach based on growth factor release is particularly promising for sporadic ALS, where there is no known gene mutation for targeted gene therapy approaches. This approach can also be used to treat retinal degeneration, regardless of specific mutations. We have shown that fNPC-GDNFs are a powerful cell and gene therapy product for several neurological disorders. But unlike fetal-derived cells, iPSC derivation from an individual’s blood or skin avoids low tissue availability and permits for personalized medicine (Svendsen, 2013). Furthermore, while fetal-derived transplants in the brain may not require long-term immunosuppression (Mendez et al., 2005), patient-specific iPSCs could avoid side effects that can arise from even short-term immunosuppression. We now have developed human iPSCs that stably produce GDNF as a promising future cell and gene therapy.

Though senesce *in vitro* can be disadvantageous as fNPCs cannot be banked indefinitely (Wright et al., 2006), reduced proliferation over time *in vivo* is advantageous to reduce tumorigenicity potential, as we have shown in the rodent central nervous system (CNS) (Gowing et al., 2014; Ostenfeld et al., 2000) and critically in the human spinal cord up to 42 months post-transplantation (Baloh et al., 2022). In contrast, while continued proliferation with iPSCs facilitates large scale-up (Ausubel et al., 2011), a risk of tumor formation exists if pluripotent cells remain within the iNPC cultures (Yamanaka, 2020). However, this study shows that iNPC-GDNFs have no combined expression of pluripotency markers *OCT4, NANOG* and *KLF4 in vitro.* Furthermore, iNPCs are non-tumorigenic post-transplantation in this study and our previous studies (Sareen et al., 2014; Tsai et al., 2015), and we have now extended this finding to 9 months. Reduced tumorigenic potential upon lineage differentiation has also been shown with iPSC-derived cardiomyocytes compared with iPSCs (Liu et al., 2013). While fewer cells in cluster 0 suggests that iNPC-GDNF neurospheres are initially not as specified toward a glial fate as fNPCs, iNPC-GDNFs do develop into an astrocytic fate after differentiation in culture or post-transplantation, indicating cells likely had an early glial progenitor fate. After extended passaging, the iNPC-GDNF line used throughout this study remained karyotypically normal; however, one batch of cultured iNPC-GDNFs developed trisomy 12, as reported with cultured pluripotent human embryonic stem cells (Draper et al., 2004). Interestingly, we have also shown that cultured fNPCs can develop a trisomy, on chromosomes 7 and 19, but still do not develop tumors after transplantation (Sareen et al., 2009).

Human iPSC-derived neural progenitors have been previously examined by our lab and others. Our EZ sphere protocol required expansion to at least passage 10 and a complex 14-week differentiation process to generate specified neural progenitors (Ebert et al., 2013; Sareen et al., 2014; Tsai et al., 2015). The new process developed here involves a simple 10-day monolayer treatment with the dual SMAD inhibitors LDN193189 and SB431542 to efficiently generate neuroepithelial progenitor cells, which are then adapted to suspension culture and expanded. This simple and fully defined process for iNPC generation is now primed for transition to cGMP production.

Similar to iPSC-derived neural progenitor transplants in the RCS rat (Tsai et al., 2015), this study shows that iNPC-GDNFs survive and migrate extensively in the subretinal space. We have previously shown that neural progenitor transplants do not provide retinal cell replacement (Jones et al., 2016; Tsai et al., 2015). Rather, cells can differentiate into astrocytes that could improve the diseased environment. A single injection of cells protected 30% of the retina and preserved visual function. fNPCs can regulate the immune response by inhibiting microglial activation, promote antioxidant effects, phagocytose retinal pigment epithelium outer segments, and release trophic factors FGF2 and IGF-1 (Jones et al., 2016; Tsai et al., 2015). In addition to these mechanisms of action, GDNF provides direct protection of photoreceptors via GDNF receptors, as for motor neurons (Jomary et al., 2004; Trupp et al., 1996). GDNF also indirectly protects photoreceptors via GDNF receptors on retinal Müller glia (Hauck et al., 2006), which then increase production of bFGF, BDNF, and GDNF (Harada et al., 2003).

While we demonstrated EZ sphere-derived NPC engraftment in the rat spinal cord (Sareen et al., 2014), we had not demonstrated efficacy in a disease model. Here we show that iNPC-GDNFs engraft in the SOD1 rat spinal cord and protect motor neurons. Interestingly, there was a significant increase in ChAT+ neuron numbers across all size bins between transplanted and non-transplanted sides in treated animals, in contrast to fNPC-GDNF that specifically protect large motor neurons (>600 μM) in the spinal cord and motor cortex (Suzuki et al., 2007; Thomsen et al., 2018). Disparities in cell effectiveness on motor neuron survival warrant further investigation and may relate to differences in fetal and iPSC-derived NPC snRNA-seq profiles. While motor neurons were protected, neither iNPC-GDNFs in this study or fNPCs in prior studies significantly protected hindlimb motor function (Suzuki et al., 2007), which may be due to the severity of this transgenic rat model.

Motor neuron protection correlated with graft location, with the greatest protective effect achieved when the graft was most closely associated with the ventral horn. Off-target grafts did not provide improvement in motor neuron number or size, highlighting that transplant delivery and targeting are critical to achieve neuroprotection. This was evident in our phase I/IIa clinical trial delivering CNS10-NPC-GDNF to the ALS patient spinal cord, in which dorsal grafts may have contributed to cell reflux, neuroma formation at the dorsal root ganglia, pain in some participants, and lack of overall effects on motor neurons and function (Baloh et al., 2022).

iNPC-GDNFs have clear potential, though some limitations exist. Off-site or excessive GDNF levels can cause side effects in patients as well as rare cerebellar Purkinje cell loss and aberrant neuronal sprouting in animal models (Georgievska et al., 2002; Hovland et al., 2007; Nutt et al., 2003). While we confirmed GDNF is safe up to 42 months in the human spinal cord (Baloh et al., 2022), future studies should develop controllable GDNF release (Akhtar et al., 2018) to tailor GDNF transgene expression for an individualized patient dose and to permit gene shut-off in the case of severe side effects. We previously demonstrated that lenti-GDNF transduction with a viral titer similar to that used here yielded fNPCs with about two to four inserted gene copies per cell (Capowski et al., 2007; Shelley et al., 2014). While the location of GDNF transgene integration was not determined, we have extensively confirmed that transduced cells are safe following transplantation. A caveat to our studies is that RP and ALS rat models were treated before overt disease onset, so treatment still needs to be assessed at a clinically relevant stage after disease onset. Further, while iNPC-GDNFs protected motor neurons in the SOD1 rat, disease onset was not significantly delayed. Given that both spinal and cortical motor neurons die in ALS and that upper motor neurons play a critical role in disease progression (Thomsen et al., 2014), treating more diseased sites is likely required for a greater therapeutic benefit. fNPC-GDNF transplants in the ALS rat motor cortex delayed disease onset and extended lifespan (Thomsen et al., 2018), providing the basis for our current clinical trial delivering CNS10-NPC-GDNF to the motor cortex of ALS patients. Future studies should evaluate iNPC-GDNF efficacy following delivery to both the spinal cord and motor cortex in ALS rats. An *ex vivo* combined cell and gene therapy approach permits delivery of both supportive astrocytes and a protective neurotrophic factor without *in vivo* genetic manipulations (Okano, 2022a). This approach has broad utility across multiple neurodegenerative conditions and CNS injuries, highlighting the need for scalable development of cell-based therapies (Okano, 2022b).

iNPC-GDNFs can protect diseased cells and function. These cells show long-term engraftment and GDNF production, as well as differentiation into astrocytes. Grafted spinal cords showed no evidence of cancerous growths or uncontrolled cell proliferation. Long-term safety data, coupled with the very low levels of pluripotency genes expressed *in vitro*, suggests that iNPC-GDNFs are a safe, scalable, and renewable source for cell transplantation therapies. Our current efficacy and safety studies provide a strong rationale to pursue the use of iNPC-GDNFs. Generating a GMP bank of iNPC-GDNFs followed by investigational new drug-enabling safety studies with the clinical product are the next steps required to move this promising combined cell and gene therapy into clinical trials for various neurodegenerative diseases.

## Experimental procedures

### Resource availability

#### Corresponding authors

Clive Svendsen: Clive.Svendsen@cshs.org, Shaomei Wang: shaomei.wang@cshs.org.

#### Materials availability

Requests for raw and analyzed data and materials are promptly reviewed by the Cedars-Sinai Board of Governor’s Regenerative Medicine Institute to verify if the request is subject to any intellectual property or confidentiality obligations. Patient-related data not included in the paper may be subject to patient confidentiality. Any data and materials that can be shared will be released via a material transfer agreement.

### Ethics statement

All cell lines and protocols in the present study were used in accordance with guidelines approved by the Stem Cell Research Oversight committee and institutional review board under the auspice institutional review board and Stem Cell Research Oversight protocols Pro00032834 (iPSC Core Repository and Stem Cell Program) and Pro00021505 (Svendsen Stem Cell Program). All animal work was approved and performed under the guidelines of the Cedars-Sinai Medical Center Institutional Animal Care and Use Committee under protocols 8517 for spinal cord and 7611 for retina. All animals in retinal studies were treated in accordance with the ARVO Statement for the Use of Animals in Ophthalmic and Vision Research.

### Cell culture

The iPSC line (named CS02iCTR-Tn11) was generated by the Cedars-Sinai iPSC Core using previous protocols (Barrett et al., 2014). iPSCs were maintained in E8 medium on Matrigel and passaged every 5 days using Versene. iPSCs at passage 17–35 were used. For differentiation, iPSCs at ∼80% confluency were singularized with Accutase and plated onto Matrigel-coated six-well plates at 200,000 cells per cm^2^ in E8 medium with 5 μM Y27632. Cultures were induced toward a neural progenitor cell using dual SMAD inhibition with differentiation media (supplemental experimental procedures for full details) for 10 days with daily media changes. Cultures were then treated with Versene and gently lifted with a cell scraper into SEFL media (Stemline with EGF, FGF2, LIF and heparin) (supplemental experimental procedures for full details). Aggregates were transferred to a poly-HEMA-coated T25-flask to establish neurospheres. Differentiated iNPCs were expanded in SEFL media as neurospheres in poly-HEMA-coated tissue culture flasks for up to 30 passages. Using a McIlwain tissue chopper (Shelley et al., 2014; Svendsen et al., 1998) or in-house mesh chopping device, spheres were passaged weekly. iNPCs were transduced with lentivirus GDNF (0.125 pg of p24 per cell) to create the iNPC-GDNFs as reported (Capowski et al., 2007; Shelley et al., 2014). iNPC-GDNF neurospheres were collected at passage 29, dissociated with TrypLE, resuspended in cell freezing medium, and cryopreserved.

### Immunocytochemistry

Cryopreserved vials containing single-cell suspensions of fNPC-GDNFs or iNPC-GDNFs were plated on poly-l-ornithine-treated Matrigel-coated glass coverslips. Cells were differentiated in Stemline medium with 2% B27 supplement for 7 days, with medium exchange every third day. Cells were fixed in 4% paraformaldehyde (PFA), washed in phosphate buffered saline (PBS), permeabilized in 1% Triton X-100 in PBS, and stained overnight at 4°C with GFAP and S100β antibodies in 5% normal donkey serum, 0.125% Triton X-100 and PBS. Samples were washed in PBS and stained with secondary antibodies for 2 h at room temperature, followed by a DAPI nuclear counterstain. Images were acquired using a Leica DM6000B microscope with a 20x objective.

### Single-nuclei RNA sequencing

Nuclei were isolated from fNPC-GDNF and iNPC-GDNF neurospheres. For single-nuclei library preparation and sequencing, the standard 10x protocol was used per the "Chromium NextGEM Single Cell 3' Reagent Kits v3.1 User Guide, Rev D." See supplemental experimental procedures for full details.

### Animals

#### RCS rats

Pigmented dystrophic RCS rats (*rdy*^*+*^*, p*^*+*^) (n = 13) received subretinal injection of 2 μL of iNPC-GDNFs at 15,000 cells/μL in balanced salt solution (BSS) at postnatal day (P) 21–23. The fellow eye served as the control, with either BSS injection (sham, n = 4) or no treatment (n = 9) with our published protocol (Tsai et al., 2015). Animals received daily intraperitoneal injection of dexamethasone for 2 weeks (1.6 mg/kg per day) post-surgery and were administered cyclosporine A in the drinking water (210 mg/L). Animals were euthanized at P90 by CO_2_ inhalation. RCS rats were tested by OKR and ERG at approximately P60 and P90, per our published protocols (Gamm et al., 2007; Wang et al., 2008). See supplemental experimental procedures for full details.

#### SOD1^G93A^ rats

At day 70–95, male rats (n = 10) were injected with 5,000 iNPC-GDNF cells/μl at 2 μL per site, into three sites spaced 2 mm apart in the left lumbar spinal cord (X = 0.75 mm, Z = 1.65 mm), along with untreated rats (n = 9) as controls. Rats were immunosuppressed with daily intraperitoneal injections of cyclosporine (10 mg/kg). Rats were euthanized at disease onset, defined by two consecutive BBB scores ≤15, by transcardial perfusion with 4% PFA. Body weight was monitored weekly. A modified BBB locomotor test (Basso et al., 1995) assessed hindlimb function. Kaplan-Meier was performed to assess onset.

#### Nude rats

Male immunodeficient athymic nude rats (n = 10) (Charles River) at ∼100 days were injected with 50,000 iNPC-GDNF cells/μl as above. Animals were euthanized at 3 months post-transplantation (n = 1) to confirm engraftment and 9 months (n = 9), by transcardial perfusion.

#### Retinal and spinal cord analysis

Retinal sections were processed as published (Tsai et al., 2015). See supplemental experimental procedures for full details. The length of ONL protection was measured on cresyl violet-stained retinal montage sections (more than two layers of ONL, six retinas, six sections/retina) against the whole retinal length using Java-based image processing software (ImageJ; National Institutes of Health, Bethesda, MD). Spinal cords were processed as previously described (Gowing et al., 2014). See supplemental experimental procedures for full details.

#### ChAT+ cell quantification

Four sections adjacent to identified graft sites and co-labeled for SC121 and ChAT+ host motor neurons were imaged at 10x using the Leica DFC365 FX camera, Leica DM6000 B microscope, and Leica Application Suite Advanced Fluorescence 3.2.0.9652 program. Images from treated animals (n = 8, average of at least eight sections per animal) and untreated animals (n = 6, average of four sections per animal) were used for motor neuron counts using the Freehand Selections tool and Region of Interest Manager in ImageJ. Images also underwent automated size analysis of motor neuron areas using IMARIS software with manual thresholding. Untreated (n = 2) and treated (n = 2) animals were removed from the IMARIS analysis due to insufficient contrast in the ChAT staining for the software to accurately distinguish positive staining from background.

#### Regressive H&E (nude rats)

For hematoxylin and eosin (H&E) staining, mounted sections were dried and then defatted and rehydrated in dH_2_O. Sections were stained with hematoxylin for 10 min and washed in running tap water. Slides were dipped 5–10 times in 1% HCl in 70% EtOH, then washed in running tap water and dipped in 0.5% lithium carbonate in ammonia water (0.1%) for 30 s. Sections were washed in tap water and stained with eosin (diluted 1:5 in dH_2_O) for 5–10 s. Slides were then dehydrated in 95% EtOH, three changes of 100% EtOH, three changes of Xylene, and coverslipped with mounting media.

### Statistics

Use of statistical tests is described in legends. GraphPad Prism 9 software was used for all calculations. Error bars represent standard deviations. Statistical tests include one-way ANOVA with Tukey’s HSD, unpaired t test with Welch’s correction, and multiple paired t tests with Holm-Šídák correction. Survival curves were compared by Log rank Mantel-Cox test (chi square 0.6011, df 1, p value 0.4381) and Gehan-Breslow-Wilcoxon test (chi square 0.7974, df 1, p value 0.3719). Significance is considered at p < 0.05.

## Author contributions

Conceptualization: A.H.L., P.A., S.W., and C.N.S. Methodology: A.H.L., A.M., P.A., V.J.G., S.B., R.H., G.L., K.R., O.S., B.L., S.R., S.W., and C.N.S. Investigation: A.H.L., A.M., S.W., and C.N.S. Visualization: A.H.L., A.M., A.W., A.F., S.R., K.R., S.B., V.J.G., S.S, S.W., and C.N.S. Funding acquisition: C.N.S. Project administration: S.W. and C.N.S. Supervision: A.H.L., A.M., S.W., and C.N.S. Writing: A.H.L., A.M., S.S., S.W., and C.N.S.

## Data Availability

All transcriptomic data in this study are available in the GEO repository: GSE214210. R code is available on GitHub: https://github.com/shaughnmb/2022_laperle_et_al.

## Supplemental information

Document S1. Figures S1–S4, Table S1, and supplemental experimental procedures

Document S2. Article plus supplemental information
