# Supplementary material for: Human iPSC-derived neural progenitor cells secreting GDNF provide protection in rodent models of ALS and retinal degeneration
Source: Stem Cell Reports. 2023 Apr 20;18(8):1629–42. doi: 10.1016/j.stemcr.2023.03.016 (PMC10444557; doi:10.1016/j.stemcr.2023.03.016)
Supplement: Document S1. Figures S1–S4, Table S1, and supplemental experimental procedures [file mmc1.pdf]

Stem Cell Reports, Volume *18*

Supplemental Information

Human iPSC-derived neural progenitor cells secreting GDNF provide protection in rodent models of ALS and retinal degeneration

Alexander H. Laperle, V. Alexandra Moser, Pablo Avalos, Bin Lu, Amanda Wu, Aaron Fulton, Stephany Ramirez, Veronica J. Garcia, Shaughn Bell, Ritchie Ho, George Lawless, Kristina Roxas, Saba Shahin, Oksana Shelest, Soshana Svendsen, Shaomei Wang, and Clive N. Svendsen

Supplemental Materials

Supplemental Figures

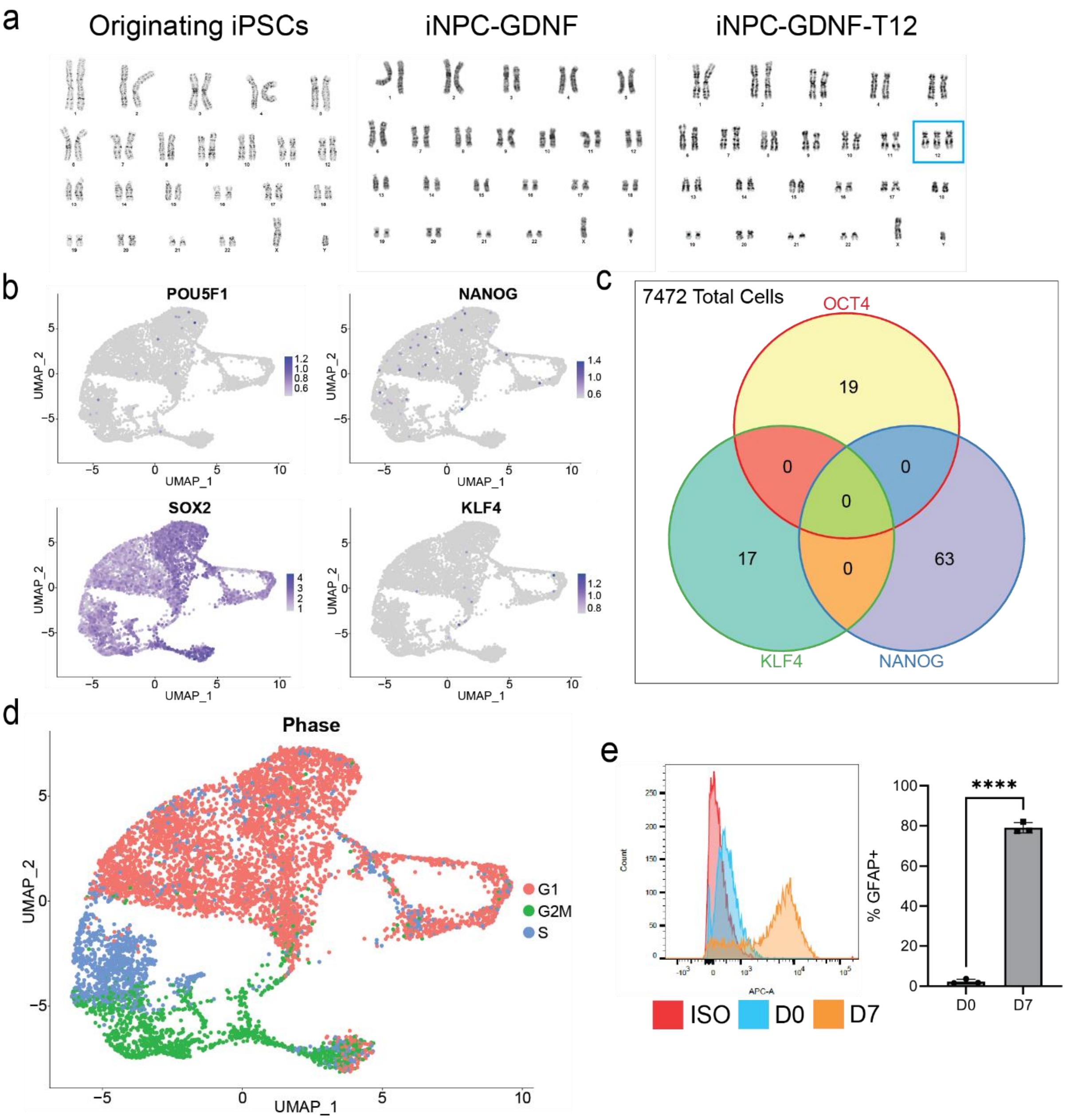

Figure S1: Extended iNPC-GDNF characterization, related to Figure 1: (a) G-band karyotype of originating iPSCs and two iNPC-GDNF batches. iNPC-GDNF-WT retained a normal karyotype while iNPC-GDNF-T12 developed nearly 100% trisomy of chromosome 12 (blue box). (b) Feature plots show expression of pluripotency genes. (c) Venn diagram of cells expressing multiple pluripotency genes. (d) snRNA-seq clustering grouped by cell cycle. (e) Histograms comparing GFAP levels at D0 and D7, with a control of isotype-stained cells (ISO), and quantification of % GFAP+ cells at D0 and D7. **** $p<0.0001$ via unpaired t test with Welch's correction.

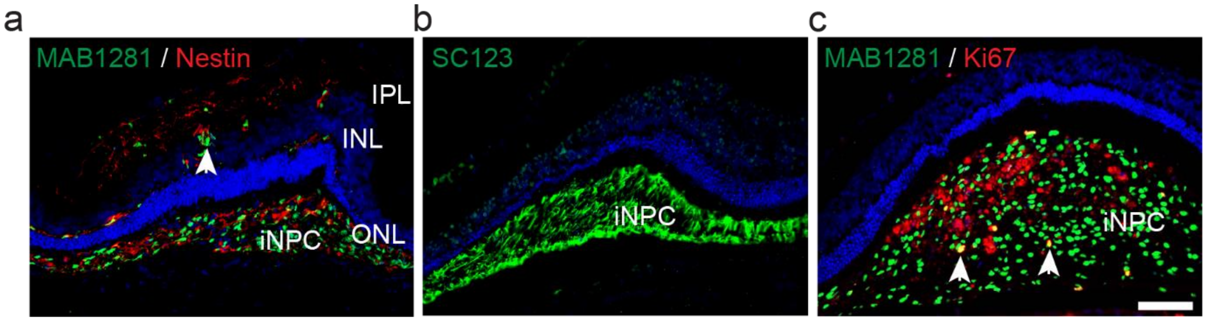

Figure S2: Extended characterization of iNPC-GDNFs in the RCS rat eye, related to Figure 2. Grafted iNPC-GDNFs from D90 RCS rat eyes were positive for MAB1281 and human nestin (a) and SC123 (b). (c) MAB1281 and Ki67 staining shows minimal double-positive cells (arrowheads). Scale bar = 75 μm. INL: Inner nuclear layer, IPL: Inner plexiform layer, ONL: Outer nuclear layer.

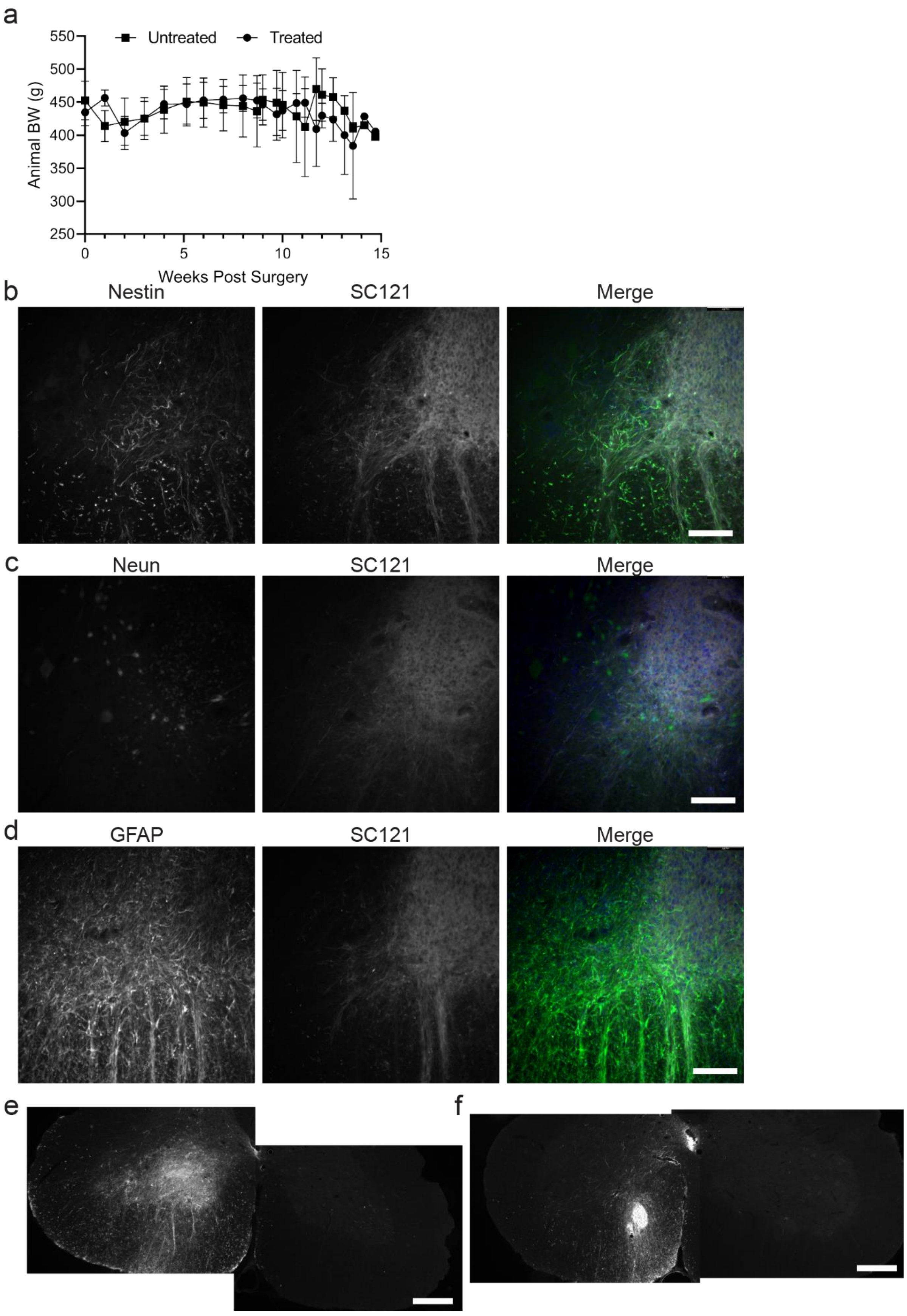

a
Untreated
Treated
Animal BW (g)
550
500
450
400
350
300
250
0
5
10
15
Weeks Post Surgery
b
Nestin
SC121
Merge
c
Neun
SC121
Merge
d
GFAP
SC121
Merge
e
f

Figure S3: Extended characterization of iNPC-GDNFs in the SOD1^{G93A} rat spinal cord, related to Figure 3. (a) Body weight (grams) of all animals after transplantation are similar between treated and untreated rats. Immunofluorescence showing iNPC-GDNFs stained with SC121 along with (b) Nestin, (c) Neun, and (d) GFAP. Immunohistochemistry showing SC121 in the spinal cord of (e) animal #239 (highest treated limb BBB score at onset) and (f) animal #240 (lowest treated hindlimb BBB score at onset). Scale bars = 100 µm (b-d) and 250 µm (e,f).

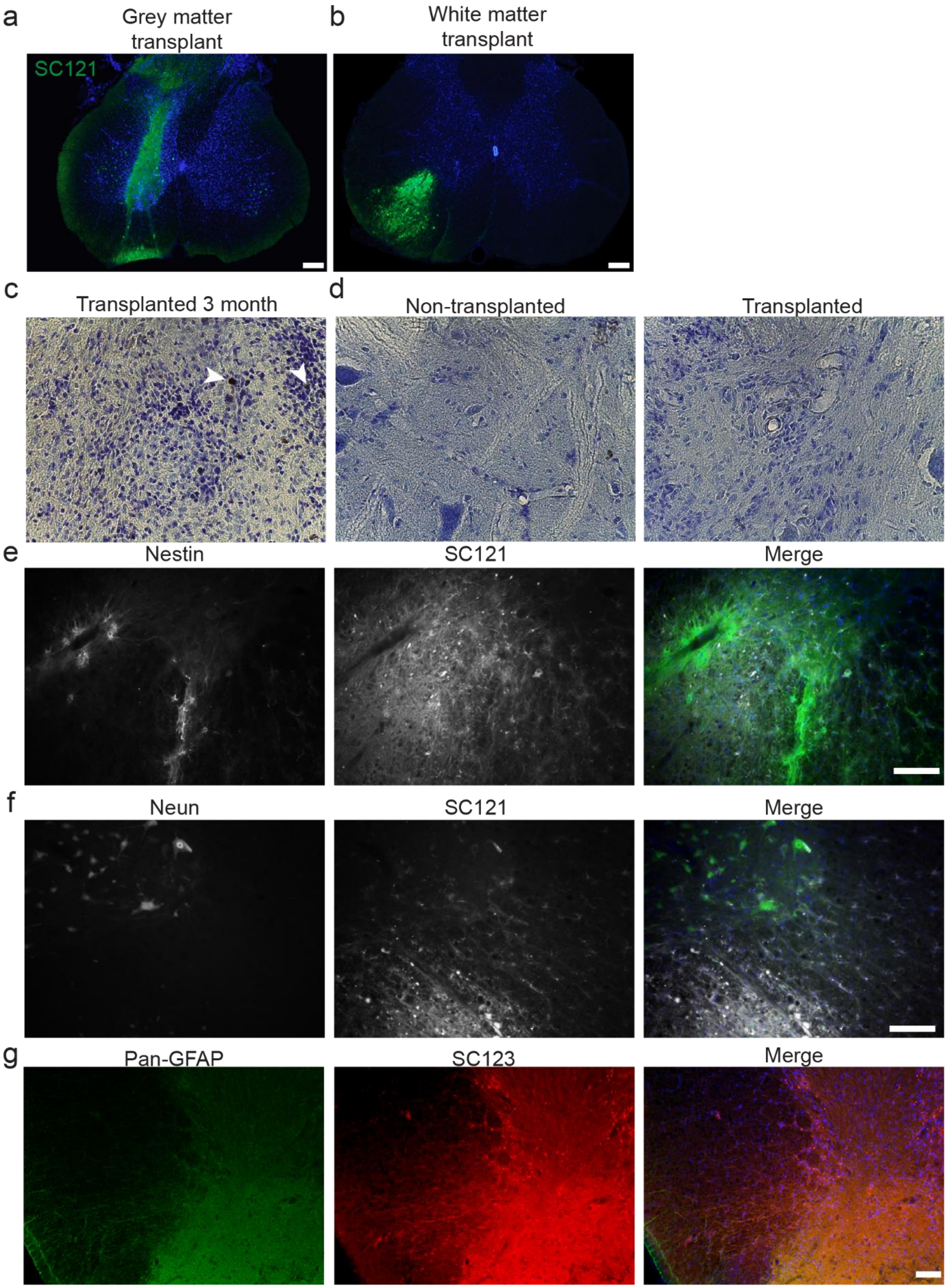

a
Grey matter transplant
SC121
b
White matter transplant
c
Transplanted 3 month
d
Non-transplanted
Transplanted
e
Nestin
SC121
Merge
f
Neun
SC121
Merge
g
Pan-GFAP
SC123
Merge

Figure S4: Extended characterization of long-term iNPC-GDNF grafts in the nude rat spinal cord, related to Figure 4. Grafts at 9 months stained for (a) SC121. Ki67 immunohistochemistry with H&E counterstain of grafts at (b) 3- and (c) 9-months post-transplantation. White arrows indicate examples of positive cells. Immunofluorescence showing iNPC-GDNF stained with SC121 along with (e) Nestin and (f) Neun. (g) pan-GFAP and human-specific GFAP (SC123). Scale bars = 250 µm (a,b) and 100 µm (e-g).

Supplemental Table

Cluster	fNPC-GDNF	iNPC-GDNF
0	95%	5%
1	33%	67%
2	0%	100%
3	36%	64%
4	1%	99%
5	37%	63%
6	52%	48%
7	94%	6%
8	100%	ND
9	53%	47%
10	100%	ND

Table S1: snRNA-seq cluster composition, related to Figure 1. Percent contribution of each cluster by cell source**.**

Supplemental Experimental Procedures

Materials

Reagent	Manufacturer	Catalogue #
E8 media	Produced in house	N/A
Versene	Thermo Fisher Scientific	15040066
Accutase	Sigma	SCR005
PS TC plates	Fisher	08-772-1B
Y27632	Stemgent	04-0012-02
DMEM/F12	Thermo Fisher Scientific	11320082
Neurobasal	Thermo Fisher Scientific	21103049
N2	Thermo Fisher Scientific	17502048
B27-vitamin A	Thermo Fisher Scientific	12587010
MEM Non-Essential Amino Acids Solution	Thermo Fisher Scientific	11140050
L-glutamine	Thermo Fisher Scientific	25030081
Human insulin	Thermo Fisher Scientific	12585014
LDN-193189	Stemgent	04-0074-02
SB431542	Cayman Chemicals	13031
Stemline media	Sigma	S3194
Human EGF	Peprotech	AF-100-15
Human FGF2	Peprotech	100-18B
Human LIF	Sigma	LIF1010
Heparin	Sigma	H3149
Poly-HEMA	Thermo Fisher Scientific	P3932
PS TC Flasks	Thermo Fisher Scientific	29185-308
TrypLE	Thermo Fisher Scientific	12604013
cGMP-grade lenti-SIN-WP-mPGK-GDNF	Indiana University	N/A
Cell freezing medium	Sigma	C6295
poly-l-ornithine (PLO)	Sigma	P4638
Matrigel	Corning	354230
Paraformaldehyde (PFA)	Fisher	50-980-495
Phosphate buffered saline (PBS)	Fisher	MT21031CV
Triton X-100	Fisher	AAA16046AP
Tris buffered saline (TBS)	Bio-Rad	1706435
Normal horse serum	Vector	S-2000-20
Normal donkey serum	Sigma	566460
Bovine serum albumin	Sigma	A9418
Vectashield mounting medium	Vector	H-1000-10
Hematoxylin	Sigma	HHS3201L
Lithium carbonate	Rowley Biochemicals	K-680-1
0.1% ammonia water	Electron Microscopy Sciences	26698-02
Eosin	Sigma	HT110332
Permanent mounting media	Thermo Fisher Scientific	4112

Antibodies

Application	Antibody	Manufacturer	Catalogue #	Dilution
ICC	GFAP	Dako	Z0334	1:2000
ICC	S100β	Sigma	S2532-.2ML	1:1000
ICC, IHC	Alexa Fluor 488	Invitrogen	A21206	1:500
ICC, IHC	Alexa Fluor 594	Invitrogen	A21203	1:500
Flow	Alexa Fluor 647	Invitrogen	A32733	1:500
ICC, IHC	49,69-diamidino-2-phenylindole (DAPI)	Thermo Fisher Scientific	D1306	300nM
IHC	Cone Arrestin	Millipore	AB15282	1:10,000
IHC	Synaptophysin	Millipore	573822	1:2,000
IHC	Human Nuclei	Millipore	MAB1281	1:300
IHC	Human Nestin (eye)	Millipore	ABD69	1:2,000
IHC	Human Nestin (spinal cord)	Millipore	ABD69	1:500
IHC	GFAP (eye)	Millipore	SAB2107063	1:1,000
IHC	GFAP (spinal cord)	Dako	Z0334	1:2000
IHC	Ki67 (eye)	Millipore	AB9260	1:500
IHC	Ki67 (spinal cord)	Thermo Fisher Scientific	RM-9106-S	1:200
IHC	SC123	Takara Bio	Y40420	1:500
IHC	SC121 (spinal cord)	Takara Bio	Y40410	1:50
IHC	GDNF (spinal cord)	R&D Systems	BAF212	1:50
IHC	ChAT	R&D Systems	AF3447	1:100
IHC	IBA1	Novus Biologicals	NB100-1028	1:300
IHC	Neun	Cell Signaling	24307	1:250
IHC	Biotinylated Secondaries	Vector	Vector, BA-9500 or BA-1100	1:200
IHC	Avidin-Biotin Complex	Vector	PK-4000	N/A
IHC	DAB kit	Vector	SK-4100	N/A
IHC	Alexa Fluor 488 (anti Goat)	Invitrogen	A11055	1:500
Flow	GFAP	Dako	Z0334	1:250
Flow	Rabbit isotype control	Cell Signaling	3900S	1:250

Cell culture media formulations

Differentiation Media	Reagent
50%	DMEM/F12
50%	Neurobasal
1%	N2
2%	B27-vitamin A
1%	MEM Non-Essential Amino Acids Solution
2mM	L-glutamine
5µg/mL	human insulin
1µM	LDN-193189
2µM	SB431542

SEFL Media	Reagent
Full Volume	Stemline media
100ng/mL	human EGF
100ng/mL	human FGF2
10ng/mL	human LIF
5µg/mL	Heparin

Single Nuclei RNA Sequencing

Nuclei isolation: Nuclei isolation was based on a modified published protocol (Corces et al., 2017). All steps were performed on ice. Frozen cell pellets were thawed on ice, resuspended in cold homogenization buffer (5mM $MgCl_2$, 3mM $Mg(Ac)_2$, 10mM Tris pH7.8, 0.017 mM PMSF, 0.17 mM β-mercaptoethanol (β-Me), 320 mM Sucrose, 0.1 mM EDTA, 0.1% NP40, RNAse inhibitors, and Protease inhibitors), and transferred to a glass dounce. Cells were manually lysed. Homogenate was filtered using 70 um Flowmi pipette tip filters and gently mixed 1:1 with a 50% Iodixanol solution (5mM $MgCl_2$, 3mM $Mg(Ac)_2$, 10mM Tris pH7.8, 0.017 mM PMSF, 0.17 mM β-Me, RNAse inhibitors, Protease inhibitors, and 50% Iodixanol). A centrifugation gradient was set up using 600 μl 40% Iodixanol solution (5mM $MgCl_2$, 3mM $Mg(Ac)_2$, 10mM Tris pH7.8, 0.017 mM PMSF, 0.17 mM β-Me, RNAse inhibitors, Protease inhibitors, 160 mM Sucrose, 0.2 μg/μl OrangeG, and 40% Iodixanol), 600 μl 29% Iodixanol solution (5mM $MgCl_2$, 3mM $Mg(Ac)_2$, 10mM Tris pH7.8, 0.017 mM PMSF, 0.17 mM β-Me RNAse inhibitors, Protease inhibitors, 160 mM Sucrose, and 29% Iodixanol) and 800 μl of the 50% Iodixanol + Sample mixture (25% Iodixanol final concentration). This was centrifuged at 3000 x g for 1 hour with breaks disengaged. Any debris on the surface was removed, and 200 μl of the thin cloudy layer containing the nuclei was extracted, mixed with 1.8 ml PBS + 1% BSA solution, and centrifuged at 500 x g for 10 minutes with breaks engaged. 1.8 ml supernatant was removed and the remaining 200 μl plus nuclei pellet was resuspended in 1.8 ml PBS + 1% BSA solution. The nuclei suspension was counted via hemocytometer and checked for nucleus quality. A portion of this suspension was diluted to the correct nuclei concentration for the 10x Chromium NextGEM 3' protocol with a target of ~4,000 nuclei per sample.

Single nuclei library preparation and sequencing: The standard 10x protocol was used per the "Chromium NextGEM Single Cell 3' Reagent Kits v3.1 User Guide, Rev D" (single index). Briefly, nuclei were resuspended in the master mix and loaded together with partitioning oil and gel beads into the chip to generate the gel bead-in-emulsion (GEM). The poly-A RNA from the nucleus lysate contained in every single GEM was retrotranscripted to cDNA, which contains an Ilumina R1 primer sequence, Unique Molecular Identifier (UMI) and the 10x Barcode. The pooled, barcoded cDNA was then cleaned up with Silane DynaBeads, amplified by PCR, and the appropriately sized fragments were selected with SPRIselect reagent for subsequent library construction. The amplified cDNA and sequencing libraries were quality checked using an Agilent 2100 BioAnalyzer (Agilent Technologies, Santa Cruz CA) using a High Sensitivity DNA chip. Barcoded sequencing libraries were quantified by qPCR on a QuantStudio12k Flex (Thermo Fisher Scientific, Waltham, MA) system using the Collibri Library Quantification Kit (Thermo Fisher Scientific). The uniquely indexed libraries were pooled at equal ratio and sequenced on a NovaSeq 6000 (Illumina, San Diego, CA), with a sequencing depth of ~50,000 reads/cell. Raw sequencing data were demultiplexed and converted to FASTQ format using bcl2fastq v2.20.

Single nuclei data analysis: Demultiplexed fastq files were run via CellRanger v6.1.2 using the "cellranger count" command with the "--include-introns" option using the precompiled CellRanger human reference sequence “v2020-A” provided on the 10x Genomics website. CellRanger output “filtered_feature_bc_matrix files” (barcodes.tsv.gz, features.tsv.gz & matrix.mtx.gz) were loaded into R (v4.2.1, https://www.R-project.org/) via Rstudio (v2022.07.1, Build 554) and combined into one matrix file per sample with genes as rows (with both gene symbol and Ensmbl ID) and cells (nuclei) as columns. Each matrix file was then processed by summing all Ensmbl IDs with the same gene symbol to the gene symbol level via standard R matrix processing functions. The gene-summed matrices were loaded into the Seurat R package (v4.1.1) keeping all genes that

were expressed in more than one cell (Hao et al., 2021). All samples were merged into a single Seurat object (7,959 nuclei) and filtered by removing all cells with counts, genes, mitochondrial gene percentage, and ribosomal gene percentage outside of three standard deviations from the mean (7,472 nuclei). The samples were then split and normalized, and variable features were identified. Integration features were then selected and used to scale the data and run principal component analysis (PCA). Integration anchors were identified via the Seurat function "FindIntegrationAnchors()", and these anchors were used to integrate the samples into a single Seurat object. The integrated object was scaled again to account for the scale of the whole data set. PCA was run on the integrated object using 50 principal components (PCs). The number of "meaningful PCs" used in subsequent analysis was determined by comparing the actual contributed variance of each PC versus the hypothetical situation where each PC would contribute equally to the variance. 13 PCs contributed more variance than the calculated hypothetical equal-variance level and were considered "meaningful". These 13 PCs were used in the subsequent analysis. Next, the nuclei were clustered using the Seurat functions "RunUMAP()", "FindNeighbors()", and "FindClusters()". A resolution factor of 0.5 identified 10 distinct clusters. For generation of UMAP plots, the RNA slot in the integrated Seurat object was processed with the Seurat functions "NormalizeData()", "FindVariableFeatures()", and "ScaleData()" using all genes present in the data set for the "features" option of "ScaleData()". The Venn diagram was generated using the "Venn()" function in the Vennerable R package (https://github.com/js229/Vennerable).

Flow cytometry: Cells from 3 cryopreserved vials of iNPC-GDNFs were split at thaw with half fixed immediately in 4% paraformaldehyde (PFA) (D0) and half plated for 7 days (D7) for differentiation as a monolayer culture. Monolayer grown cells were washed once in phosphate buffered saline (PBS) then 1mL Accutase was added to each well and incubated for 5 mins at 37°C. Singularized cells were collected and pelleted by centrifugation (1500 RPM for 3 mins). Cells were gently resuspended in 4% PFA in PBS and allowed to fix for 10-15 mins at room temperature. All fixed single cells were permeabilized using 1% Triton X-100 and stained using primary antibodies against GFAP or an isotype control (1:250) overnight at 4 °C. Samples were washed 3 times in PBS with 0.1% TritonX-100 and 5% NDS. Secondary antibodies (Alexa Fluor 647) were used at 1:500 at room temperature for one hour. Stained samples were quantified on an LSR Fortessa cytometer using BD FACSDiva software and analyzed using FloJo software.

Animals: Given our extensive knowledge that neural progenitor cells alone are less effective than cells producing GDNF, and to uphold the 3Rs to "replace, reduce and refine" animal use (Hubrecht and Carter, 2019), studies did not use iNPC alone rat cohorts. Animals were housed and maintained at the Cedars-Sinai Department of Comparative Medicine vivarium. All animal studies were approved and supervised by the Institutional Animal Care and Use Committee and all animals were treated in accordance with the ARVO Statement for the Use of Animals in Ophthalmic and Vision Research.

Visual function testing: All rats were tested by optokinetic response (OKR) and electroretinography (ERG) at approximately P60 and P90, according to our published protocols (Gamm et al,. 2007; Wang et al., 2008). Animals were tested for spatial visual acuity using an optometry testing apparatus. This device consists of a rotating cylinder covered with a vertical sine wave grating presented in virtual three-dimensional (3-D) space on four computer monitors arranged in a square. Unrestrained rats were placed on a platform in the center of the square, where they tracked the grating with reflexive head movements. The spatial frequency of the grating was clamped at the viewing position by repeatedly re-centering the 'cylinder' on the head of the test subject. Acuity was quantified by increasing the spatial frequency of the grating using

a psychophysics staircase progression until the optokinetic reflex was lost, thereby obtaining a maximum threshold. Dark adapted full field ERG responses were recorded following our published protocol (Tsai et al., 2015). Special care was taken to maintain electrode placement in a consistent position in all animals. Averages of 3–5 traces (set 2 minutes apart to ensure recovery of rod responsiveness) were obtained.

Histology and Immunohistochemistry

RCS model: Eyes from the RCS rats were removed and post-fixed in 4% PFA for 1-2 hour, then placed in 10%, 20% sucrose for 1 hour, and placed in 30% sucrose at 4 °C overnight. The corneas and lenses were removed, and the eyes were embedded in optimal cutting temperature compound (OCT). Sectioning was performed on a cryostat and retinal sections were collected in 5 series according to our previous protocol (Tsai et al., 2015). One of every five slides was stained with cresyl violet (CV) and the remaining slides were stored at −80 °C for antibody staining. CV-stained images were acquired with a regular bright field microscope and ProgRes capture system (Jenoptik, Jupiter, FL). Retinal sections were immunohistochemically stained according to our published protocol (Tsai et al., 2015).

Quantification of outer nuclear layer: ONL thickness and donor cell distribution were measured on cresyl violet-stained sections (6 retinas, 6 sections/retina). Retinal montage sections were prepared for measuring the length of the preserved ONL as compared to the whole retinal length. Measurements were made using Java-based image processing software (ImageJ; National Institutes of Health, Bethesda, MD).

SOD1^{G93A} and nude rat models: Spinal cords were removed, post-fixed in 4% PFA for 24 h, and transferred to 30% sucrose until the tissue sank. The lumbar region was then sectioned at 35 μm sections using a microtome. Sections were washed 3 times in PBS for 5 min, quenched in 3% hydrogen peroxide/3% methanol for 10 min, then washed 3 times in 0.5% Triton X-100 in 1X Tris buffered saline (TBS). Sections were then blocked in 5% Normal Horse Serum and 2% Bovine Serum Albumin for 60 mins at room temperature, followed by overnight incubation at 4ºC in primary antibodies. Sections were washed with 0.2% Triton X-100 in 1X TBS 3 times for 10 min, then placed into the appropriate biotinylated or fluorescent secondary antibodies for 1.5 to 2 hours at room temperature. Sections with immunofluorescent staining were washed 3 times for 5 min in 1X TBS, incubated with DAPI for 2 minutes, and washed again with 1X TBS 3 times for 5 min each before being mounted onto slides and coverslipped using Vectashield mounting medium. Sections incubated with biotinylated secondary antibodies were placed in Avidin-Biotin Complex for 1 hour at room temperature and then washed 3 times for 10 min each in 0.2% Triton X-100 in 1X TBS. A DAB kit was used for chromogenic detection with 3,3′-Diaminobenzidine. Sections were then washed twice for 5 min each in 1X TBS, mounted onto slides, dehydrated, and coverslipped with permanent mounting media.

Supplemental References

Corces, M.R., Trevino, A.E., Hamilton, E.G., Greenside, P.G., Sinnott-Armstrong, N.A., Vesuna, S., Satpathy, A.T., Rubin, A.J., Montine, K.S., Wu, B., et al. (2017). An improved ATAC-seq protocol reduces background and enables interrogation of frozen tissues. Nat. Methods *14*, 959–962.

Hao Y., Hao S., Andersen-Nissen E., Mauck W.M. 3rd, Zheng S., Butler A., Lee M.J., Wilk A.J., Darby C., Zager M., et al. (2021) Integrated analysis of multimodal single-cell data. Cell. *184*, 3573–3587.e29.

Hubrecht, R.C., and Carter, E. (2019). The 3rs and humane experimental technique: implementing change. Animals (Basel) *9*.
