# Supplementary material for: Human iPSC-derived neural progenitor cells secreting GDNF provide protection in rodent models of ALS and retinal degeneration
Source: Stem Cell Reports. 2023 Apr 20;18(8):1629–42. doi: 10.1016/j.stemcr.2023.03.016 (PMC10444557; doi:10.1016/j.stemcr.2023.03.016)
Supplement: Document S2. Article plus supplemental information [file mmc2.pdf]

Stem Cell Reports
Article
OPEN ACCESS

Human iPSC-derived neural progenitor cells secreting GDNF provide protection in rodent models of ALS and retinal degeneration

Alexander H. Laperle,[1,2] V. Alexandra Moser,[1,2] Pablo Avalos,[1] Bin Lu,[1] Amanda Wu,[1] Aaron Fulton,[1] Stephany Ramirez,[1] Veronica J. Garcia,[1] Shaughn Bell,[1] Ritchie Ho,[1] George Lawless,[1] Kristina Roxas,[1] Saba Shahin,[1] Oksana Shelest,[1] Soshana Svendsen,[1] Shaomei Wang,[1,*] and Clive N. Svendsen[1,*]
[1]Cedars-Sinai Board of Governors Regenerative Medicine Institute, Cedars-Sinai Medical Center, Los Angeles, CA, USA
[2]These authors contributed equally
*Correspondence: clive.svendsen@cshs.org (C.N.S.), shaomei.wang@cshs.org (S.W.)
https://doi.org/10.1016/j.stemcr.2023.03.016

SUMMARY

Human induced pluripotent stem cells (iPSCs) are a renewable cell source that can be differentiated into neural progenitor cells (iNPCs) and transduced with glial cell line-derived neurotrophic factor (iNPC-GDNFs). The goal of the current study is to characterize iNPC-GDNFs and test their therapeutic potential and safety. Single-nuclei RNA-seq show iNPC-GDNFs express NPC markers. iNPC-GDNFs delivered into the subretinal space of the Royal College of Surgeons rodent model of retinal degeneration preserve photoreceptors and visual function. Additionally, iNPC-GDNF transplants in the spinal cord of SOD1^{G93A} amyotrophic lateral sclerosis (ALS) rats preserve motor neurons. Finally, iNPC-GDNF transplants in the spinal cord of athymic nude rats survive and produce GDNF for 9 months, with no signs of tumor formation or continual cell proliferation. iNPC-GDNFs survive long-term, are safe, and provide neuroprotection in models of both retinal degeneration and ALS, indicating their potential as a combined cell and gene therapy for various neurodegenerative diseases.

INTRODUCTION

Several neurodegenerative diseases, including Parkinson's and Alzheimer's, and amyotrophic lateral sclerosis (ALS), involve unique genetic and environmental risk factors, while others such as retinitis pigmentosa (RP) involve various genetic mutations. Replacing the damaged cell population is one treatment approach; however, transplanted cells do not readily form new long-distance synaptic connections with their targets or integrate with secondary retinal neurons in the adult environment. Therefore, protection of remaining host cells is a more practical approach and suggests the need for a protective intervention that is beneficial across various neurodegenerative diseases.

ALS involves progressive motor neuron death in the spinal cord and motor cortex, leading to paralysis and death, typically within 2–5 years of diagnosis (Hulisz, 2018). There are currently no effective treatments. As glial cells are compromised in ALS, one neuroprotective strategy is to provide new glial support cells (Bruijn et al., 1997; Lepore et al., 2008). These can be derived from the human fetal forebrain and then sorted to isolate glial-restricted progenitors (GRPs) (Lepore et al., 2011; Sandrock et al., 2010). Alternatively, we have shown that human cortical fetal-derived neural progenitor cells (fNPCs) can be expanded in culture as free-floating spheres (Svendsen et al., 1998) and can differentiate into astrocytes *in vitro* and *in vivo* (Das et al., 2016; Gowing et al., 2014; Svendsen et al., 1997). While GRP and fNPC transplants survive in the spinal cord of the well-characterized SOD1^{G93A} (hereto, SOD1) transgenic rodent model of ALS, neither benefit motor neuron survival nor functional measures. This suggests that an additional strategy may be required for neuronal protection. As glial cell line-derived neurotrophic factor (GDNF) has protective effects on dopamine and motor neurons (Henderson et al., 1994; Lin et al., 1993; Zurn et al., 1994), we have genetically engineered fNPCs to stably secrete GDNF (fNPC-GDNF) (Capowski et al., 2007). Unlike fNPCs alone, this combined cell and growth factor *ex vivo* therapy could protect spinal motor neurons in the SOD1 ALS rat, as well as dopamine neurons in a Parkinsonian rat model (Behrstock et al., 2006; Klein et al., 2005; Suzuki et al., 2007). In addition, delivery of fNPCs releasing GDNF to the ALS rat motor cortex protected motor neurons, slowed disease progression, and extended lifespan (Thomsen et al., 2018). RP involves progressive loss of photoreceptors, ultimately leading to blindness. Using the well-established Royal College of Surgeons (RCS) rat model of retinal degeneration, we have shown that fNPCs can protect vision and photoreceptors and that GDNF-secreting fNPCs provide enhanced protection (Gamm et al., 2007).

We previously expanded a single fetal cortical sample under current Good Manufacturing Practice (cGMP) to derive a neural progenitor cell line, termed CNS10-NPC, which was lentivirally transduced to stably produce GDNF and banked as a clinical product (termed CNS10-NPC-GDNF) (Shelley et al., 2014). This product was used for a first-in-man cell and gene therapy for ALS with delivery to the lumbar spinal cord in a recently completed

This is an open access article under the CC BY license (http://creativecommons.org/licenses/by/4.0/).

phase I/IIa safety trial (clinical trials.gov: NCT02943850). The primary endpoint of safety at 1 year was met, with no negative effect of the transplant on motor function, and grafts survived and produced GDNF for up to 42 months post-transplantation (Baloh et al., 2022). CNS10-NPC-GDNF is now being delivered to the motor cortex of ALS patients in a phase I/IIa clinical trial (NCT05306457). Finally, a current phase I/IIa clinical trial is transplanting CNS10-NPC into the subretinal space of RP patients (NCT04284293).

Fetal-derived NPCs and their derivatives are a promising treatment for Parkinson's disease, ALS, and RP, as well as other neurological conditions such as Huntington's disease, stroke, and dementia (Andres et al., 2011; Goldberg et al., 2017; McBride et al., 2004). However, this product has some limitations, including low fetal tissue availability that can hinder large scale-up, thus restricting phase III trials and commercialization. Our lab and others have shown that induced pluripotent stem cells (iPSCs) provide a renewable, scalable, and safe cell source for deriving potential cell products (Rosati et al., 2018; Sareen et al., 2014; Tsai et al., 2015). Peripheral blood mononuclear cells (PBMCs) from an individual's blood sample can be reprogrammed into iPSCs, which proliferate in culture, differentiate into multiple tissue types, and can be cryopreserved and thawed (Barrett et al., 2014). We have demonstrated that human iPSCs cultured in specified media could generate neural-specific cultures that were propagated as spheres (termed EZ spheres), which could engraft and differentiate into astrocytes in the rodent spinal cord and retina (Ebert et al., 2013; Sareen et al., 2014; Tsai et al., 2015).

Here, we developed and tested an iPSC-based therapy as an alternative to fetal-derived products. We generated iPSC-derived NPCs (termed iNPCs), which were transduced to express GDNF (iNPC-GDNF), characterized, and compared with fNPC-GDNFs. iNPC-GDNFs were transplanted into the subretinal space of RCS rats where they protected photoreceptors and vision, the spinal cord of SOD1 rats where they protected motor neurons, and the spinal cord of nude rats where they showed long-term survival and safety. Based on safety and efficacy, iNPC-GDNFs can be pursued as a promising combined cell and gene therapy for multiple neurodegenerative diseases.

RESULTS

iNPC-GDNFs and derived astrocytes resemble fNPC-GDNFs and derived astrocytes

NPCs derived from a single human fetal cortex were maintained as free-floating spheres (termed neurospheres), expanded by mechanical passage using a tissue chopper (Shelley et al., 2014; Svendsen et al., 1998), transduced with lentivirus to stably express GDNF (Capowski et al., 2007; Shelley et al., 2014), then propagated and banked under cGMP as the CNS10-NPC-GDNF clinical cell lot, and fNPC-GDNFs were banked as a research-grade cell lot used in this study. To produce iPSC-derived NPCs similar to these fNPCs, a new and substantially shorter protocol was developed. An iPSC line was generated by nucleofecting healthy PBMCs with nonintegrating oriP/EBNA1 plasmids, which allowed for episomal expression of reprogramming factors (Barrett et al., 2014). Using dual SMAD inhibition (Chambers et al., 2009), a monolayer of neuroepithelial progenitors was efficiently generated, which were then collected and transitioned into neurospheres in the presence of epidermal growth factor (EGF), fibroblast growth factor 2 (FGF2), and leukemia inhibitory factor (LIF) (Figure 1A). iNPC neurospheres were expanded by mechanical passage using a tissue chopper or mesh chopper. After passage 16, iNPCs were dissociated to single cells and lentivirally infected to stably express GDNF. Transduced cells reformed as iNPC-GDNF neurospheres that were used in the current studies.

To demonstrate consistency in the production process, two independent batches of iNPC-GDNFs were produced. G-band karyotyping revealed that one batch remained karyotypically normal, while the other batch acquired a karyotypic anomaly not present in the originating iPSC line with nearly 100% trisomy of chromosome 12, termed iNPC-GDNF-T12 (Figure S1A). To avoid complications that could arise from karyotypic abnormality, subsequent studies were performed with the karyotypically normal iNPC-GDNF batch, which remained normal up to 19 passages. To identify any potential remaining pluripotent cells in the differentiated batches, pluripotency genes *POU5F1 (OCT4), NANOG, SOX2,* and *KLF4* were plotted from single-nuclei RNA sequencing (snRNA-seq) data (Figure S1B). While individual cells expressed some of these markers at low levels, no cell expressed all four or any combination of *OCT4, NANOG,* and *KLF4* (Figure S1C). As expected, *SOX2* was expressed throughout most cells (Figure S1B), as it is also a NPC marker (Zhang and Cui, 2014).

snRNA-seq was performed to assess the similarity between iNPC-GDNF and fNPC-GDNF neurosphere cultures. Following batch normalization, UMAP (uniform manifold approximation and projection) was applied to identify clusters of cell types in an unbiased manner, which identified 11 separate clusters (Figure 1B, Table S1). When split by sample source, fNPC-GDNFs were found in all clusters except for 2 and 4, which were almost exclusively iNPC-GDNFs, while clusters 8 and 10 were entirely fNPC-GDNFs (Figure 1C, Table S1). Importantly, the overlap of a population of iNPC-GDNF clusters in most fNPC-GDNF clusters demonstrates the remarkable similarity in some cell

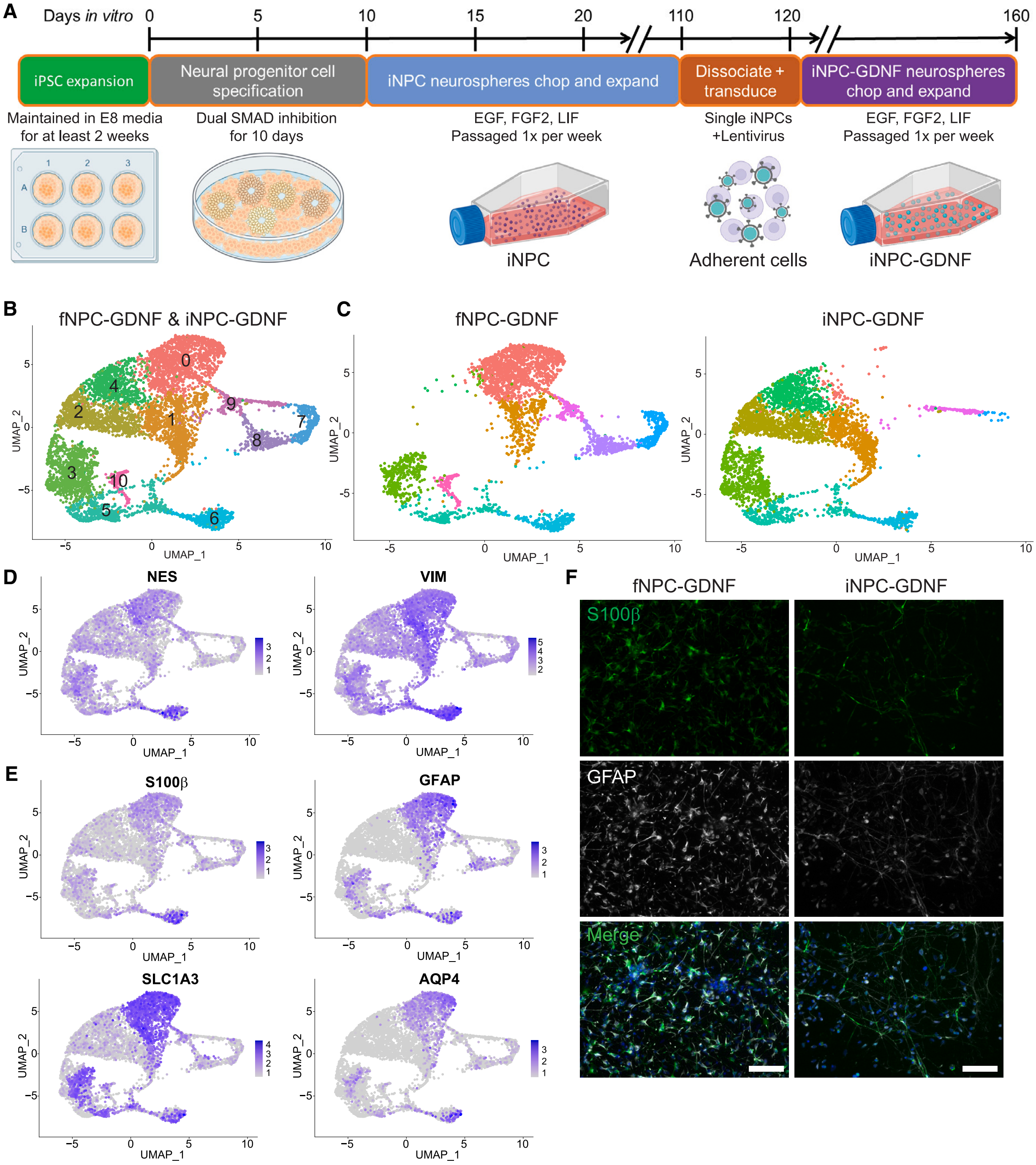

Figure 1. iNPC-GDNF and fNPC-GDNF profiles

(A) Protocol to generate and expand iNPC-GDNFs.

(B) Unbiased UMAP clustering of cells from fNPC-GDNF and iNPC-GDNF neurospheres split into 11 defined clusters.

(C) Clustering split by cell type shows that iNPC-GDNFs clustered similarly to fNPC-GDNFs in many but not all cases.

(D and E) Feature plots show expression of (D) cortical neural progenitor markers and (E) astrocyte markers.

(F) Immunocytochemistry on fNPC-GDNFs and iNPC-GDNFs differentiated for 7 days in culture shows production of S100β and GFAP, with DAPI (blue). Scale bar represents 75 μm.

See also Table S1 and Figure S1.

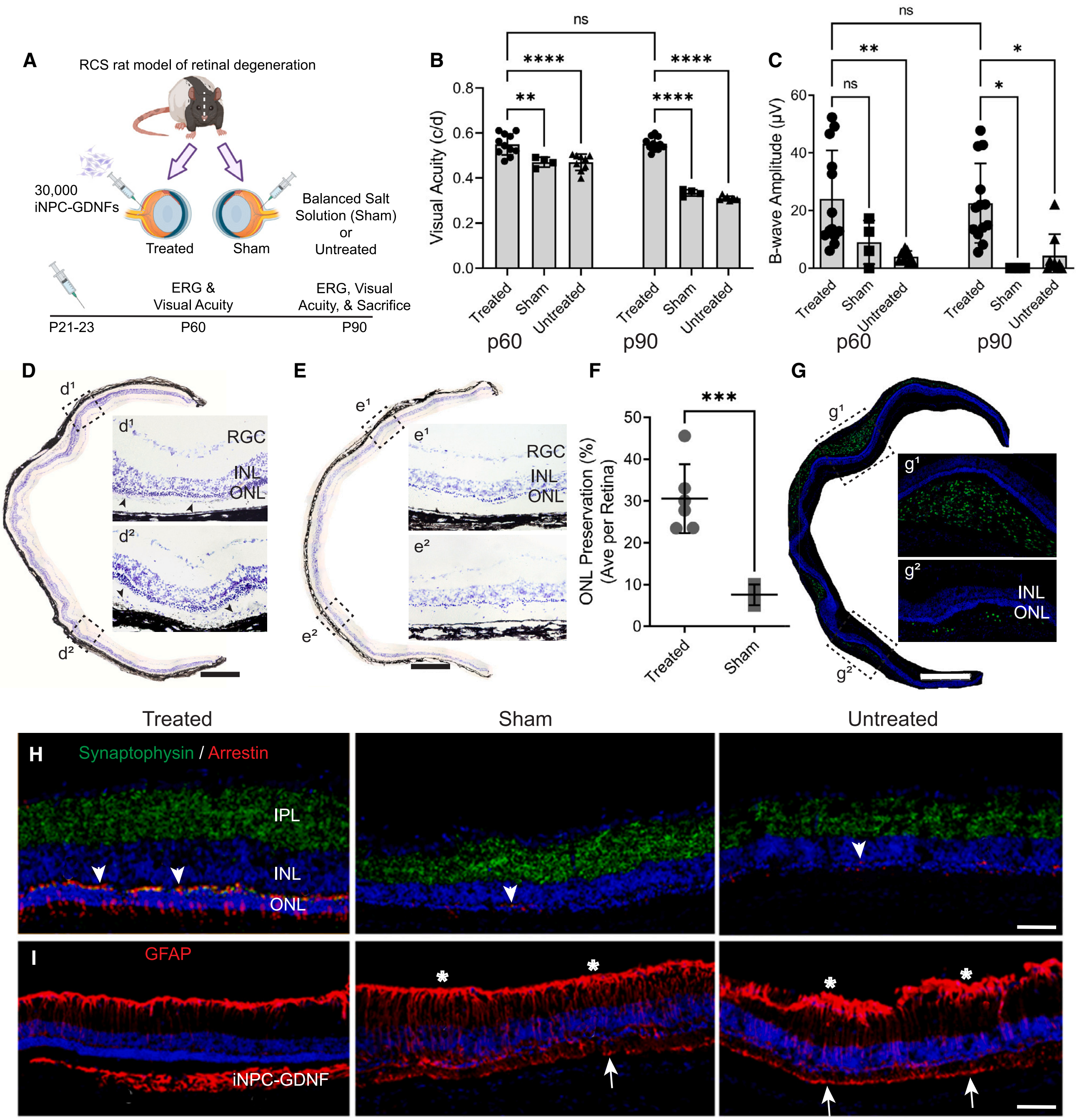

Figure 2. iNPC-GDNFs are neuroprotective in the RCS rat model of retinal degeneration

(A) Schematic of experiment RCS rats (n = 13) that received subretinal injection of iNPC-GDNFs at postnatal day (P) 21–23. The fellow eye received either balanced salt solution injection (sham, n = 4) or no treatment (n = 9).

(B and C) Visual function was evaluated by (B) OKR and (C) ERG, which showed significant preservation in cell-treated compared with control animals (****$p < 0.0001$, **$p < 0.01$, *$p < 0.05$, one-way ANOVA with Tukey's HSD, ns: not significant).

(D and E) Cresyl violet-stained retinal images show photoreceptor preservation in (D) iNPC-GDNF-treated retina with potential grafted cells (arrowheads), compared with (E) sham. d^1, d^2, e^1, and e^2 are high-power images from areas in boxed outlines.

(F) Percent of retina with at least two layers of photoreceptors in iNPC-GDNF-treated vs. sham (***$p < 0.001$ via unpaired t test with Welch's correction).

(legend continued on next page)

populations. Indeed, markers of cortical NPCs were highly expressed throughout all cells in this analysis (Figure 1D). Exclusively iNPC-GDNF clusters tended to express markers for the immature progenitors (*VIM*, *SOX2*, *NESTIN*). Interestingly, clustering was often driven by cell cycle phase (Figure S1D), again indicating strong similarities between cell types, as this distinction does not commonly appear in more diverse datasets. Cluster 0 cells showed high expression of astrocyte-related genes (Figure 1E). Given that this cluster had a low contribution in iNPC-GDNF neurospheres (Table S1), we wanted to assess their differentiation potential. iNPC-GDNFs and fNPC-GDNFs were differentiated as monolayer cultures for 7 days, with subsequent immunocytochemistry showing that both produced the astrocyte markers glial fibrillary acidic protein (GFAP) and S100β (Figure 1F). Flow cytometry further demonstrated that 80% of differentiated iNPC-GDNFs acquired GFAP production (Figure S1E). Collectively, results demonstrate that while some differences exist in fetal- and iPSC-derived NPCs, there also are substantial similarities.

iNPC-GDNFs preserve vision and photoreceptors in the RCS rat model

Fetal- and iPSC-derived NPCs can protect vision following a single subretinal injection into the RCS rat (Gamm et al., 2007; Tsai et al., 2015; Wang et al., 2008). Importantly, GDNF-secreting fNPCs provide enhanced vision protection, and GDNF delivery can preserve photoreceptors (Gamm et al., 2007; García-Caballero et al., 2018). To evaluate the efficacy of iNPC-GDNFs, RCS rats at postnatal day (P) 21–23 received a single unilateral subretinal injection of cells, and the fellow eye served as the control, with balanced salt solution (sham) injection or no treatment (Figure 2A).

Visual acuity measured by the optokinetic response (OKR) showed that sham-treated and untreated eyes had a significant decrease in visual acuity from P60 to P90 (Figure 2B). However, there was almost complete rescue with iNPC-GDNF treatment, with visual acuity over time remaining around 0.55 cycle/degree (Figure 2B), comparable to wild-type rats (McGill et al., 2007). Electroretinography (ERG), measuring the average response of the whole retina to light stimulation, revealed that iNPC-GDNF-treated eyes had significantly higher b-wave amplitude compared with controls at P60 and P90 (Figure 2C). ERG does show visual impairment compared with a wild-type rat, as ERG was compromised already when treatment started. But, critically, iNPC-GDNF treatment provided sustained protection of both ERG and OKR over the study duration.

To assess protection of retinal morphology at P90, the length of preserved photoreceptors was measured against the whole length of cresyl violet-stained retinal sections, which showed that iNPC-GDNF treatment preserved three to six photoreceptor layers, compared with most controls with only one layer remaining (Figures 2D and 2E). Quantifying the outer nuclear layer (ONL) showed that iNPC-GDNFs protected an average of 30.59% ± 3.6% of the retina, indicating roughly one-third of the retinal sections retained at least two layers of photoreceptors (Figure 2F). Further, staining with the human-specific nuclear antibody MAB1281 confirmed iNPC-GDNFs had robust survival and extensive migration in the subretinal space (Figure 2G) and, notably, demonstrated multiple layers of photoreceptors associated with donor cells at the injection site (Figure $2G^1$) and with migrating donor cells (Figure $2G^2$). iNPC-GDNF treatment also preserved cone photoreceptors, revealed by immunofluorescence with a cone arrestin antibody that showed inner and outer segments and dense cone pedicles (Figure 2H). In contrast, controls had only weak staining for cones, with no visible segments and cone pedicles. Retinal connections, based on wide and disperse synaptophysin staining, were evident in cell--treated retinas, compared with narrow staining in controls (Figure 2H). Strong GFAP staining in control retinas indicates a reactive Müller glia response (Figure 2I). This was substantially reduced in the treated retina and mainly located in the optic nerve fiber layer, suggesting that cell treatment reduced Müller glia activation. The pan-GFAP antibody also stained grafted iNPC-GDNFs, indicating differentiation into astrocytes.

Immunofluorescence with human-specific antibodies for Nestin and astrocytes (SC123) was used to characterize grafted iNPC-GDNFs, which showed that cells remained as neural progenitors or differentiated into astrocytes (Figures S2A and S2B). Consistent with our prior study (Gamm et al., 2007), a few grafted cells migrated into the inner retina, but most formed a layer of cells or lump

(G) Staining with human nuclear marker MAB1281 shows extensive distribution of grafted iNPC-GDNFs; g^1 and g^2 are high-power images from areas in boxed outlines.
(H) Synaptophysin and cone arrestin staining show cones with segments and pedicles (arrowheads) were preserved in cell-treated retina compared with controls.
(I) GFAP staining shows reactive Müller glia in controls and cell-treated retina, GFAP labels iNPC-GDNFs, and host Müller glia (stars, arrows point to Müller glial end feet). Scale bars represent (D, E, and G) 500 μm and (H and I) 75 μm. INL, inner nuclear layer; IPL, inner plexiform layer; ONL, outer nuclear layer. d^1, e^1, and g^1 indicate regions near injection site, d^2, e^2, and g^2 indicate regions distal from injection site. See also Figure S2.

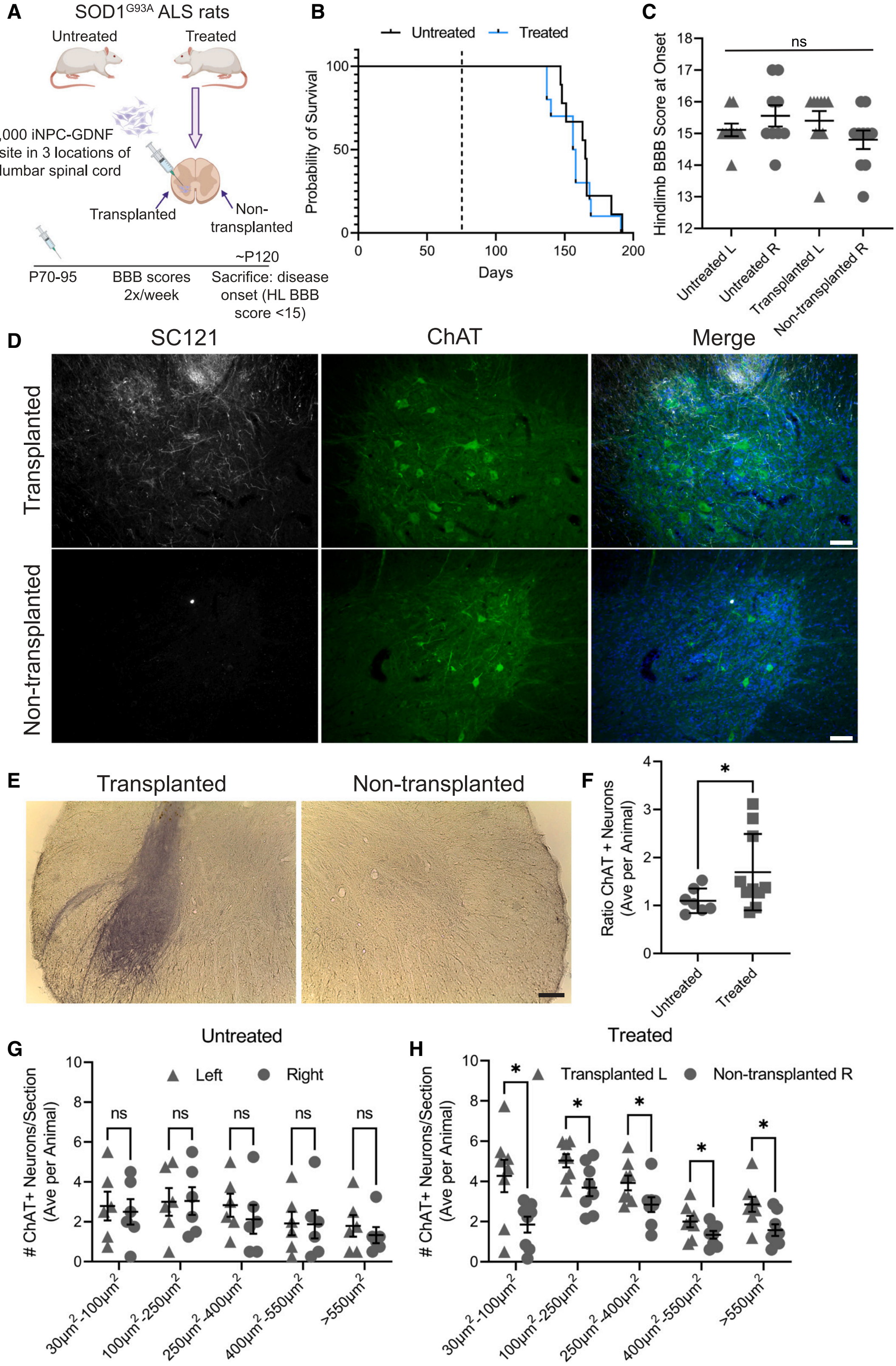

(legend on next page)

within the subretinal space. While Ki67 staining indicates proliferating host cells in the degenerative environment, only a few Ki67-positive human cells demonstrated the limited proliferation of transplanted iNPC-GDNFs (Figure S2C).

iNPC-GDNFs are neuroprotective in SOD1 ALS rat

To evaluate whether iNPC-GDNFs are protective in SOD1 ALS rats, cells were injected unilaterally into the lumbar spinal cord, with untreated SOD1 rats serving as controls (Figure 3A). Treated and untreated groups had similar body weights over the study course (Figure S3A). Basso, Beattie, and Bresnahan (BBB) locomotor scores were collected weekly, starting 1 week before transplantation, to assess hindlimb function and disease onset. There was no significant difference in BBB scores for transplanted/non-transplanted hindlimbs in treated and untreated rats, indicating no delay in disease onset (Figures 3B and 3C).

To assess iNPC-GDNF survival and host motor neurons, rats were euthanized at disease onset, and tissue was analyzed by immunohistochemistry. Grafted cells stained positive for the human-specific cytoplasmic marker (SC121) with some cells co-staining for the neural progenitor marker Nestin (Figure S3B). Minimal co-staining of SC121 with the neuronal marker Neun (Figure S3C) coupled with extensive co-staining for GFAP (Figure S3D) indicate a primarily glial identity of the grafted cells. SC121 staining confirmed iNPC-GDNFs in the treated spinal cord, but not the non-treated side, with engraftment around choline-o-acetyl transferase (ChAT)-positive host motor neurons (Figure 3D). Robust GDNF production was seen in the iNPC-GDNF-treated spinal cord but not in the non-transplanted side (Figure 3E). While all transplanted animals showed surviving grafts, the location and extent varied (Figures S3E and S3F), and it was observed that better graft targets correlated with greater motor neuron counts. To assess whether iNPC-GDNFs protected host motor neurons, spinal cord sections containing SC121+ grafts were quantified for ChAT+ motor neuron numbers and size. ImageJ analysis revealed a significantly elevated ratio of total number of motor neurons (transplanted/non-transplanted) in treated compared with untreated animals (Figure 3F). We have previously shown that fNPC-GDNF specifically protect large motor neurons (<600 μM) in the ALS rat spinal cord and motor cortex (Suzuki et al., 2007; Thomsen et al., 2018). Here, the number of ChAT+ motor neuron across all size bins was significantly increased between transplanted and non-transplanted sides in treated animals, with no difference across size bins between the left and right spinal cord in untreated animals (Figures 3G and 3H). This indicates that iNPC-GDNFs provide neuroprotection of motor neurons in the ALS rat spinal cord.

iNPC-GDNFs show long-term survival and safety in the nude rat spinal cord

To examine iNPC-GDNF long-term survival and safety, cells were transplanted unilaterally into the lumbar spinal cord of athymic nude rats (Figure 4A). These rats have a spontaneous mutation in the *Foxn1* gene and thus lack T cells, rendering them immunodeficient to enable xenograft survival without immunosuppression. iNPC-GDNF transplants survived well in both gray matter and white matter spinal cord regions (Figures S4A and S4B) and produced GDNF (Figure 4B) for up to 9 months. Histopathological examination of hematoxylin and eosin staining showed no structural abnormalities indicative of cancerous growth in any section across all animals. Ki67-postive cells were observed at 3 months but not 9 months post-transplantation, indicating reduced proliferation over time (Figures S4C and S4D). At 9 months post-transplantation, some iNPC-GDNFs retained Nestin expression (Figure S4E) with relatively little overlap of Neun (Figure S4F). Strong staining from an antibody that recognizes human and rat GFAP (pan-GFAP) and from a human-specific GFAP antibody (SC123) demonstrated that cells differentiated primarily into astrocytes (Figure S4G), and higher magnification shows clear astrocyte morphology for iNPC-GDNFs co-labeled with SC121 and GFAP (Figure 4C).

Figure 3. iNPC-GDNFs are neuroprotective in the SOD1 ALS rat

(A) Schematic of experiment. 10 male SOD1 rats at 70–95 days were unilaterally transplanted (left, L) with 10,000 iNPC-GDNFs in three sites 2 mm apart in the lumbar spinal cord and compared with 9 untreated animals.

(B) Kaplan-Meier shows probability of onset times of treated vs. untreated animals are not significantly different.

(C) Hindlimb BBB scores at disease onset are not significantly different.

(D) Immunohistochemistry shows SC121+ human grafts surrounding host ChAT+ motor neurons, with DAPI (blue).

(E) GDNF staining of transplanted compared with non-transplanted spinal cord.

(F) Ratio of ChAT+ motor neurons averaged per animal (n = 7 untreated and n = 10 treated animals).

(G and H) ChAT+ neuron size in (G) untreated animals (n = 6, average of four sections per animal) and (H) treated animals (n = 8, average of at least eight sections per animal); $^*p < 0.05$ via unpaired t test with Welch's correction (F) or multiple paired t tests corrected with the Holm-Šídák method (G and H); ns: not significant. Scale bars represent (D) 75 μm and (E) 250 μm.

See also Figure S3.

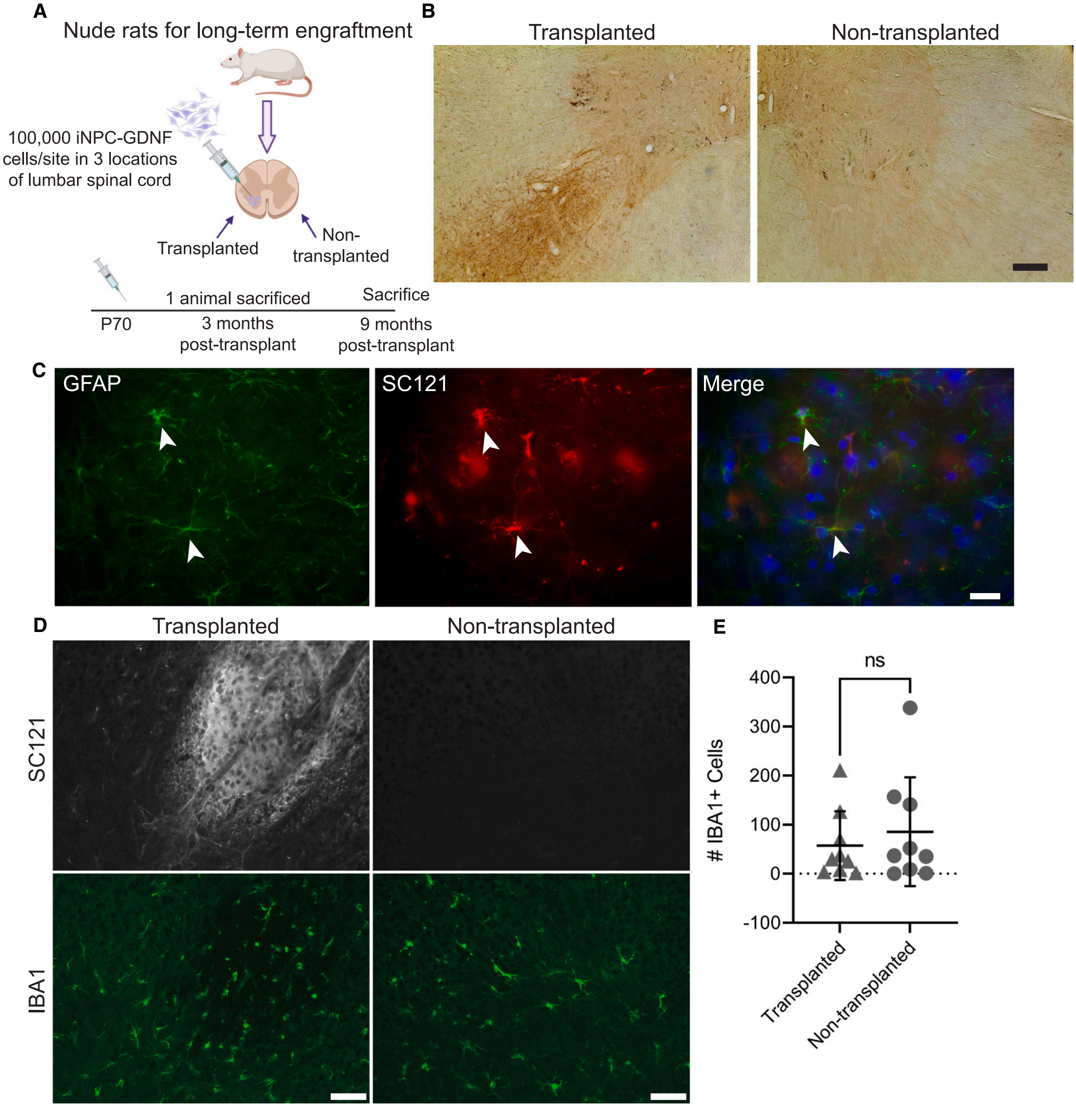

Figure 4. iNPC-GDNF spinal cord long-term grafts

(A) Schematic of experiment. Rats (n = 10) were unilaterally transplanted with 100,000 iNPC-GDNFs in three sites 2 mm apart in the lumbar spinal cord, with euthanasia at 3 months (n = 1) and 9 months (n = 9) post-transplantation.

(B and C) Grafts at 9 months were stained for (B) GDNF and (C) pan-GFAP and SC121 with DAPI (blue).

(D and E) SC121 and IBA1 staining (D) and quantification of IBA1+ cells with a paired t test (E) (average per animal, minimum of four sections analyzed per animal). ns: not significant. Scale bars represent (B) 75 μm, (C) 20 μm, and (D) 50 μm.

See also Figure S4.

To examine effects of long-term iNPC-GDNF transplants on spinal cord inflammation, immunohistochemistry was performed for human cells and the microglial marker IBA-1 (Figure 4D). While microglia number varied across animals, there was no significant increase in microglia surrounding the SC121+ graft compared with the contralateral spinal

cord (Figure 4E), indicating that iNPC-GDNFs did not enhance long-term inflammation. Collectively, results demonstrate that long-term iNPC-GDNF transplants can survive, differentiate into glia, and are safe with no evidence of tumor growth.

DISCUSSION

Neurodegeneration encompasses many different diseases and cell types with diverse genetic and environmental causes that elude pathway-targeted therapies. Instead, NPCs that engraft and differentiate into supportive glia, and which can be genetically engineered to produce a growth factor, are broadly applicable to many diseases. A focal delivery approach is critical as GDNF cannot cross the blood-brain barrier, and systemic delivery has been associated with adverse effects (Thomsen et al., 2017). A combined cell and gene therapy approach based on growth factor release is particularly promising for sporadic ALS, where there is no known gene mutation for targeted gene therapy approaches. This approach can also be used to treat retinal degeneration, regardless of specific mutations. We have shown that fNPC-GDNFs are a powerful cell and gene therapy product for several neurological disorders. But unlike fetal-derived cells, iPSC derivation from an individual's blood or skin avoids low tissue availability and permits for personalized medicine (Svendsen, 2013). Furthermore, while fetal-derived transplants in the brain may not require long-term immunosuppression (Mendez et al., 2005), patient-specific iPSCs could avoid side effects that can arise from even short-term immunosuppression. We now have developed human iPSCs that stably produce GDNF as a promising future cell and gene therapy.

Though senesce *in vitro* can be disadvantageous as fNPCs cannot be banked indefinitely (Wright et al., 2006), reduced proliferation over time *in vivo* is advantageous to reduce tumorigenicity potential, as we have shown in the rodent central nervous system (CNS) (Gowing et al., 2014; Ostenfeld et al., 2000) and critically in the human spinal cord up to 42 months post-transplantation (Baloh et al., 2022). In contrast, while continued proliferation with iPSCs facilitates large scale-up (Ausubel et al., 2011), a risk of tumor formation exists if pluripotent cells remain within the iNPC cultures (Yamanaka, 2020). However, this study shows that iNPC-GDNFs have no combined expression of pluripotency markers *OCT4, NANOG* and *KLF4 in vitro*. Furthermore, iNPCs are non-tumorigenic post-transplantation in this study and our previous studies (Sareen et al., 2014; Tsai et al., 2015), and we have now extended this finding to 9 months. Reduced tumorigenic potential upon lineage differentiation has also been shown with iPSC-derived cardiomyocytes compared with iPSCs (Liu et al., 2013). While fewer cells in cluster 0 suggests that iNPC-GDNF neurospheres are initially not as specified toward a glial fate as fNPCs, iNPC-GDNFs do develop into an astrocytic fate after differentiation in culture or post-transplantation, indicating cells likely had an early glial progenitor fate. After extended passaging, the iNPC-GDNF line used throughout this study remained karyotypically normal; however, one batch of cultured iNPC-GDNFs developed trisomy 12, as reported with cultured pluripotent human embryonic stem cells (Draper et al., 2004). Interestingly, we have also shown that cultured fNPCs can develop a trisomy, on chromosomes 7 and 19, but still do not develop tumors after transplantation (Sareen et al., 2009).

Human iPSC-derived neural progenitors have been previously examined by our lab and others. Our EZ sphere protocol required expansion to at least passage 10 and a complex 14-week differentiation process to generate specified neural progenitors (Ebert et al., 2013; Sareen et al., 2014; Tsai et al., 2015). The new process developed here involves a simple 10-day monolayer treatment with the dual SMAD inhibitors LDN193189 and SB431542 to efficiently generate neuroepithelial progenitor cells, which are then adapted to suspension culture and expanded. This simple and fully defined process for iNPC generation is now primed for transition to cGMP production.

Similar to iPSC-derived neural progenitor transplants in the RCS rat (Tsai et al., 2015), this study shows that iNPC-GDNFs survive and migrate extensively in the subretinal space. We have previously shown that neural progenitor transplants do not provide retinal cell replacement (Jones et al., 2016; Tsai et al., 2015). Rather, cells can differentiate into astrocytes that could improve the diseased environment. A single injection of cells protected 30% of the retina and preserved visual function. fNPCs can regulate the immune response by inhibiting microglial activation, promote antioxidant effects, phagocytose retinal pigment epithelium outer segments, and release trophic factors FGF2 and IGF-1 (Jones et al., 2016; Tsai et al., 2015). In addition to these mechanisms of action, GDNF provides direct protection of photoreceptors via GDNF receptors, as for motor neurons (Jomary et al., 2004; Trupp et al., 1996). GDNF also indirectly protects photoreceptors via GDNF receptors on retinal Müller glia (Hauck et al., 2006), which then increase production of bFGF, BDNF, and GDNF (Harada et al., 2003).

While we demonstrated EZ sphere-derived NPC engraftment in the rat spinal cord (Sareen et al., 2014), we had not demonstrated efficacy in a disease model. Here we show that iNPC-GDNFs engraft in the SOD1 rat spinal cord and protect motor neurons. Interestingly, there was a significant increase in ChAT+ neuron numbers across all size bins between transplanted and non-transplanted sides

in treated animals, in contrast to fNPC-GDNF that specifically protect large motor neurons (>600 μM) in the spinal cord and motor cortex (Suzuki et al., 2007; Thomsen et al., 2018). Disparities in cell effectiveness on motor neuron survival warrant further investigation and may relate to differences in fetal and iPSC-derived NPC snRNA-seq profiles. While motor neurons were protected, neither iNPC-GDNFs in this study or fNPCs in prior studies significantly protected hindlimb motor function (Suzuki et al., 2007), which may be due to the severity of this transgenic rat model.

Motor neuron protection correlated with graft location, with the greatest protective effect achieved when the graft was most closely associated with the ventral horn. Off-target grafts did not provide improvement in motor neuron number or size, highlighting that transplant delivery and targeting are critical to achieve neuroprotection. This was evident in our phase I/IIa clinical trial delivering CNS10-NPC-GDNF to the ALS patient spinal cord, in which dorsal grafts may have contributed to cell reflux, neuroma formation at the dorsal root ganglia, pain in some participants, and lack of overall effects on motor neurons and function (Baloh et al., 2022).

iNPC-GDNFs have clear potential, though some limitations exist. Off-site or excessive GDNF levels can cause side effects in patients as well as rare cerebellar Purkinje cell loss and aberrant neuronal sprouting in animal models (Georgievska et al., 2002; Hovland et al., 2007; Nutt et al., 2003). While we confirmed GDNF is safe up to 42 months in the human spinal cord (Baloh et al., 2022), future studies should develop controllable GDNF release (Akhtar et al., 2018) to tailor GDNF transgene expression for an individualized patient dose and to permit gene shut-off in the case of severe side effects. We previously demonstrated that lenti-GDNF transduction with a viral titer similar to that used here yielded fNPCs with about two to four inserted gene copies per cell (Capowski et al., 2007; Shelley et al., 2014). While the location of GDNF transgene integration was not determined, we have extensively confirmed that transduced cells are safe following transplantation. A caveat to our studies is that RP and ALS rat models were treated before overt disease onset, so treatment still needs to be assessed at a clinically relevant stage after disease onset. Further, while iNPC-GDNFs protected motor neurons in the SOD1 rat, disease onset was not significantly delayed. Given that both spinal and cortical motor neurons die in ALS and that upper motor neurons play a critical role in disease progression (Thomsen et al., 2014), treating more diseased sites is likely required for a greater therapeutic benefit. fNPC-GDNF transplants in the ALS rat motor cortex delayed disease onset and extended lifespan (Thomsen et al., 2018), providing the basis for our current clinical trial delivering CNS10-NPC-GDNF to the motor cortex of ALS patients. Future studies should evaluate iNPC-GDNF efficacy following delivery to both the spinal cord and motor cortex in ALS rats. An *ex vivo* combined cell and gene therapy approach permits delivery of both supportive astrocytes and a protective neurotrophic factor without *in vivo* genetic manipulations (Okano, 2022a). This approach has broad utility across multiple neurodegenerative conditions and CNS injuries, highlighting the need for scalable development of cell-based therapies (Okano, 2022b).

iNPC-GDNFs can protect diseased cells and function. These cells show long-term engraftment and GDNF production, as well as differentiation into astrocytes. Grafted spinal cords showed no evidence of cancerous growths or uncontrolled cell proliferation. Long-term safety data, coupled with the very low levels of pluripotency genes expressed *in vitro*, suggests that iNPC-GDNFs are a safe, scalable, and renewable source for cell transplantation therapies. Our current efficacy and safety studies provide a strong rationale to pursue the use of iNPC-GDNFs. Generating a GMP bank of iNPC-GDNFs followed by investigational new drug-enabling safety studies with the clinical product are the next steps required to move this promising combined cell and gene therapy into clinical trials for various neurodegenerative diseases.

EXPERIMENTAL PROCEDURES

Resource availability

Corresponding authors

Clive Svendsen: Clive.Svendsen@cshs.org, Shaomei Wang: shaomei.wang@cshs.org.

Materials availability

Requests for raw and analyzed data and materials are promptly reviewed by the Cedars-Sinai Board of Governor's Regenerative Medicine Institute to verify if the request is subject to any intellectual property or confidentiality obligations. Patient-related data not included in the paper may be subject to patient confidentiality. Any data and materials that can be shared will be released via a material transfer agreement.

Data and code availability

All transcriptomic data in this study are available in the GEO repository: GSE214210. R code is available on GitHub: https://github.com/shaughnmb/2022_laperle_et_al.

Ethics statement

All cell lines and protocols in the present study were used in accordance with guidelines approved by the Stem Cell Research Oversight committee and institutional review board under the auspice institutional review board and Stem Cell Research Oversight protocols Pro00032834 (iPSC Core Repository and Stem Cell Program) and Pro00021505 (Svendsen Stem Cell Program). All animal work was approved and performed under the guidelines of the Cedars-Sinai Medical Center Institutional Animal Care and Use Committee under protocols 8517 for spinal cord and 7611 for retina. All animals in retinal studies were treated in accordance

with the ARVO Statement for the Use of Animals in Ophthalmic and Vision Research.

Cell culture

The iPSC line (named CS02iCTR-Tn11) was generated by the Cedars-Sinai iPSC Core using previous protocols (Barrett et al., 2014). iPSCs were maintained in E8 medium on Matrigel and passaged every 5 days using Versene. iPSCs at passage 17–35 were used. For differentiation, iPSCs at ~80% confluency were singularized with Accutase and plated onto Matrigel-coated six-well plates at 200,000 cells per cm^2 in E8 medium with 5 μM Y27632. Cultures were induced toward a neural progenitor cell using dual SMAD inhibition with differentiation media (supplemental experimental procedures for full details) for 10 days with daily media changes. Cultures were then treated with Versene and gently lifted with a cell scraper into SEFL media (Stemline with EGF, FGF2, LIF and heparin) (supplemental experimental procedures for full details). Aggregates were transferred to a poly-HEMA-coated T25-flask to establish neurospheres. Differentiated iNPCs were expanded in SEFL media as neurospheres in poly-HEMA-coated tissue culture flasks for up to 30 passages. Using a McIlwain tissue chopper (Shelley et al., 2014; Svendsen et al., 1998) or in-house mesh chopping device, spheres were passaged weekly. iNPCs were transduced with lentivirus GDNF (0.125 pg of p24 per cell) to create the iNPC-GDNFs as reported (Capowski et al., 2007; Shelley et al., 2014). iNPC-GDNF neurospheres were collected at passage 29, dissociated with TrypLE, resuspended in cell freezing medium, and cryopreserved.

Immunocytochemistry

Cryopreserved vials containing single-cell suspensions of fNPC-GDNFs or iNPC-GDNFs were plated on poly-l-ornithine-treated Matrigel-coated glass coverslips. Cells were differentiated in Stemline medium with 2% B27 supplement for 7 days, with medium exchange every third day. Cells were fixed in 4% paraformaldehyde (PFA), washed in phosphate buffered saline (PBS), permeabilized in 1% Triton X-100 in PBS, and stained overnight at 4°C with GFAP and S100β antibodies in 5% normal donkey serum, 0.125% Triton X-100 and PBS. Samples were washed in PBS and stained with secondary antibodies for 2 h at room temperature, followed by a DAPI nuclear counterstain. Images were acquired using a Leica DM6000B microscope with a 20x objective.

Single-nuclei RNA sequencing

Nuclei were isolated from fNPC-GDNF and iNPC-GDNF neurospheres. For single-nuclei library preparation and sequencing, the standard 10x protocol was used per the "Chromium NextGEM Single Cell 3′ Reagent Kits v3.1 User Guide, Rev D." See supplemental experimental procedures for full details.

Animals

RCS rats

Pigmented dystrophic RCS rats (*rdy*$^+$, *p*$^+$) (n = 13) received subretinal injection of 2 μL of iNPC-GDNFs at 15,000 cells/μL in balanced salt solution (BSS) at postnatal day (P) 21–23. The fellow eye served as the control, with either BSS injection (sham, n = 4) or no treatment (n = 9) with our published protocol (Tsai et al., 2015). Animals received daily intraperitoneal injection of dexamethasone for 2 weeks (1.6 mg/kg per day) post-surgery and were administered cyclosporine A in the drinking water (210 mg/L). Animals were euthanized at P90 by CO_2 inhalation. RCS rats were tested by OKR and ERG at approximately P60 and P90, per our published protocols (Gamm et al., 2007; Wang et al., 2008). See supplemental experimental procedures for full details.

SOD1^{G93A} rats

At day 70–95, male rats (n = 10) were injected with 5,000 iNPC-GDNF cells/μl at 2 μL per site, into three sites spaced 2 mm apart in the left lumbar spinal cord (X = 0.75 mm, Z = 1.65 mm), along with untreated rats (n = 9) as controls. Rats were immunosuppressed with daily intraperitoneal injections of cyclosporine (10 mg/kg). Rats were euthanized at disease onset, defined by two consecutive BBB scores ≤15, by transcardial perfusion with 4% PFA. Body weight was monitored weekly. A modified BBB locomotor test (Basso et al., 1995) assessed hindlimb function. Kaplan-Meier was performed to assess onset.

Nude rats

Male immunodeficient athymic nude rats (n = 10) (Charles River) at ~100 days were injected with 50,000 iNPC-GDNF cells/μl as above. Animals were euthanized at 3 months post-transplantation (n = 1) to confirm engraftment and 9 months (n = 9), by transcardial perfusion.

Retinal and spinal cord analysis

Retinal sections were processed as published (Tsai et al., 2015). See supplemental experimental procedures for full details. The length of ONL protection was measured on cresyl violet-stained retinal montage sections (more than two layers of ONL, six retinas, six sections/retina) against the whole retinal length using Java-based image processing software (ImageJ; National Institutes of Health, Bethesda, MD). Spinal cords were processed as previously described (Gowing et al., 2014). See supplemental experimental procedures for full details.

ChAT+ cell quantification

Four sections adjacent to identified graft sites and co-labeled for SC121 and ChAT+ host motor neurons were imaged at 10x using the Leica DFC365 FX camera, Leica DM6000 B microscope, and Leica Application Suite Advanced Fluorescence 3.2.0.9652 program. Images from treated animals (n = 8, average of at least eight sections per animal) and untreated animals (n = 6, average of four sections per animal) were used for motor neuron counts using the Freehand Selections tool and Region of Interest Manager in ImageJ. Images also underwent automated size analysis of motor neuron areas using IMARIS software with manual thresholding. Untreated (n = 2) and treated (n = 2) animals were removed from the IMARIS analysis due to insufficient contrast in the ChAT staining for the software to accurately distinguish positive staining from background.

Regressive H&E (nude rats)

For hematoxylin and eosin (H&E) staining, mounted sections were dried and then defatted and rehydrated in dH_2O. Sections were stained with hematoxylin for 10 min and washed in running tap water. Slides were dipped 5–10 times in 1% HCl in 70% EtOH, then washed in running tap water and dipped in 0.5% lithium carbonate in ammonia water (0.1%) for 30 s. Sections were washed in tap water and stained with eosin (diluted 1:5 in dH_2O) for 5–10 s.

Slides were then dehydrated in 95% EtOH, three changes of 100% EtOH, three changes of Xylene, and coverslipped with mounting media.

Statistics

Use of statistical tests is described in legends. GraphPad Prism 9 software was used for all calculations. Error bars represent standard deviations. Statistical tests include one-way ANOVA with Tukey's HSD, unpaired t test with Welch's correction, and multiple paired t tests with Holm-Šídák correction. Survival curves were compared by Log rank Mantel-Cox test (chi square 0.6011, df 1, p value 0.4381) and Gehan-Breslow-Wilcoxon test (chi square 0.7974, df 1, p value 0.3719). Significance is considered at $p < 0.05$.

SUPPLEMENTAL INFORMATION

Supplemental information can be found online at https://doi.org/10.1016/j.stemcr.2023.03.016.

AUTHOR CONTRIBUTIONS

Conceptualization: A.H.L., P.A., S.W., and C.N.S. Methodology: A.H.L., A.M., P.A., V.J.G., S.B., R.H., G.L., K.R., O.S., B.L., S.R., S.W., and C.N.S. Investigation: A.H.L., A.M., S.W., and C.N.S. Visualization: A.H.L., A.M., A.W., A.F., S.R., K.R., S.B., V.J.G., S.S, S.W., and C.N.S. Funding acquisition: C.N.S. Project administration: S.W. and C.N.S. Supervision: A.H.L., A.M., S.W., and C.N.S. Writing: A.H.L., A.M., S.S., S.W., and C.N.S.

ACKNOWLEDGMENTS

This work was supported in part by grants from the California Institute for Regenerative Medicine (CIRM) DR2A-05320 to CNS and CIRM-EDUC2-12638 to SR and the Board of Governors Regenerative Medicine Institute. We thank the Cedars-Sinai Genomics Core for assistance with snRNA-seq, the Cedars-Sinai Cytogenetics Core for karyotype analysis, and Jovita Dimas-Harms and Jake Inzalaco for immunohistochemistry assistance. Schematics were created using biorender.com

CONFLICT OF INTERESTS

The authors declare no competing financial interests. Intellectual protection-patent "Cortical Neural progenitor cells from iPSCs" No. 62/924,523 was filed October 22, 2019.

Received: November 7, 2022
Revised: March 24, 2023
Accepted: March 27, 2023
Published: April 20, 2023

REFERENCES

Akhtar, A.A., Gowing, G., Kobritz, N., Savinoff, S.E., Garcia, L., Saxon, D., Cho, N., Kim, G., Tom, C.M., Park, H., et al. (2018). Inducible expression of GDNF in transplanted iPSC-derived neural progenitor cells. Stem Cell Rep. *10*, 1696–1704.

Andres, R.H., Horie, N., Slikker, W., Keren-Gill, H., Zhan, K., Sun, G., Manley, N.C., Pereira, M.P., Sheikh, L.A., McMillan, E.L., et al. (2011). Human neural stem cells enhance structural plasticity and axonal transport in the ischaemic brain. Brain *134*, 1777–1789.

Ausubel, L.J., Lopez, P.M., and Couture, L.A. (2011). GMP scale-up and banking of pluripotent stem cells for cellular therapy applications. Methods Mol. Biol. *767*, 147–159.

Baloh, R.H., Johnson, J.P., Avalos, P., Allred, P., Svendsen, S., Gowing, G., Roxas, K., Wu, A., Donahue, B., Osborne, S., et al. (2022). Transplantation of human neural progenitor cells secreting GDNF into the spinal cord of patients with ALS: a phase 1/2a trial. Nat. Med. *28*, 1813–1822.

Barrett, R., Ornelas, L., Yeager, N., Mandefro, B., Sahabian, A., Lenaeus, L., Targan, S.R., Svendsen, C.N., and Sareen, D. (2014). Reliable generation of induced pluripotent stem cells from human lymphoblastoid cell lines. Stem Cells Transl. Med. *3*, 1429–1434.

Basso, D.M., Beattie, M.S., and Bresnahan, J.C. (1995). A sensitive and reliable locomotor rating scale for open field testing in rats. J. Neurotrauma *12*, 1–21.

Behrstock, S., Ebert, A., McHugh, J., Vosberg, S., Moore, J., Schneider, B., Capowski, E., Hei, D., Kordower, J., Aebischer, P., and Svendsen, C.N. (2006). Human neural progenitors deliver glial cell line-derived neurotrophic factor to parkinsonian rodents and aged primates. Gene Ther. *13*, 379–388.

Bruijn, L.I., Becher, M.W., Lee, M.K., Anderson, K.L., Jenkins, N.A., Copeland, N.G., Sisodia, S.S., Rothstein, J.D., Borchelt, D.R., Price, D.L., and Cleveland, D.W. (1997). ALS-linked SOD1 mutant G85R mediates damage to astrocytes and promotes rapidly progressive disease with SOD1-containing inclusions. Neuron *18*, 327–338.

Capowski, E.E., Schneider, B.L., Ebert, A.D., Seehus, C.R., Szulc, J., Zufferey, R., Aebischer, P., and Svendsen, C.N. (2007). Lentiviral vector-mediated genetic modification of human neural progenitor cells for ex vivo gene therapy. J. Neurosci. Methods *163*, 338–349.

Chambers, S.M., Fasano, C.A., Papapetrou, E.P., Tomishima, M., Sadelain, M., and Studer, L. (2009). Highly efficient neural conversion of human ES and iPS cells by dual inhibition of SMAD signaling. Nat. Biotechnol. *27*, 275–280.

Das, M.M., Avalos, P., Suezaki, P., Godoy, M., Garcia, L., Chang, C.D., Vit, J.-P., Shelley, B., Gowing, G., and Svendsen, C.N. (2016). Human neural progenitors differentiate into astrocytes and protect motor neurons in aging rats. Exp. Neurol. *280*, 41–49.

Draper, J.S., Smith, K., Gokhale, P., Moore, H.D., Maltby, E., Johnson, J., Meisner, L., Zwaka, T.P., Thomson, J.A., and Andrews, P.W. (2004). Recurrent gain of chromosomes 17q and 12 in cultured human embryonic stem cells. Nat. Biotechnol. *22*, 53–54.

Ebert, A.D., Shelley, B.C., Hurley, A.M., Onorati, M., Castiglioni, V., Patitucci, T.N., Svendsen, S.P., Mattis, V.B., McGivern, J.V., Schwab, A.J., et al. (2013). EZ spheres: a stable and expandable culture system for the generation of pre-rosette multipotent stem cells from human ESCs and iPSCs. Stem Cell Res. *10*, 417–427.

Gamm, D.M., Wang, S., Lu, B., Girman, S., Holmes, T., Bischoff, N., Shearer, R.L., Sauvé, Y., Capowski, E., Svendsen, C.N., and Lund, R.D. (2007). Protection of visual functions by human neural progenitors in a rat model of retinal disease. PLoS One *2*, e338.

García-Caballero, C., Lieppman, B., Arranz-Romera, A., Molina-Martínez, I.T., Bravo-Osuna, I., Young, M., Baranov, P., and Herrero-Vanrell, R. (2018). Photoreceptor preservation induced by intravitreal controlled delivery of GDNF and GDNF/melatonin in rhodopsin knockout mice. Mol. Vis. *24*, 733–745.

Georgievska, B., Kirik, D., and Björklund, A. (2002). Aberrant sprouting and downregulation of tyrosine hydroxylase in lesioned nigrostriatal dopamine neurons induced by long-lasting overexpression of glial cell line derived neurotrophic factor in the striatum by lentiviral gene transfer. Exp. Neurol. *177*, 461–474.

Goldberg, N.R.S., Marsh, S.E., Ochaba, J., Shelley, B.C., Davtyan, H., Thompson, L.M., Steffan, J.S., Svendsen, C.N., and Blurton-Jones, M. (2017). Human neural progenitor transplantation rescues behavior and reduces α-synuclein in a transgenic model of dementia with lewy bodies. Stem Cells Transl. Med. *6*, 1477–1490.

Gowing, G., Shelley, B., Staggenborg, K., Hurley, A., Avalos, P., Victoroff, J., Latter, J., Garcia, L., and Svendsen, C.N. (2014). Glial cell line-derived neurotrophic factor-secreting human neural progenitors show long-term survival, maturation into astrocytes, and no tumor formation following transplantation into the spinal cord of immunocompromised rats. Neuroreport *25*, 367–372.

Harada, C., Harada, T., Quah, H.M.A., Maekawa, F., Yoshida, K., Ohno, S., Wada, K., Parada, L.F., and Tanaka, K. (2003). Potential role of glial cell line-derived neurotrophic factor receptors in Müller glial cells during light-induced retinal degeneration. Neuroscience *122*, 229–235.

Hauck, S.M., Kinkl, N., Deeg, C.A., Swiatek-de Lange, M., Schöffmann, S., and Ueffing, M. (2006). GDNF family ligands trigger indirect neuroprotective signaling in retinal glial cells. Mol. Cell Biol. *26*, 2746–2757.

Henderson, C.E., Phillips, H.S., Pollock, R.A., Davies, A.M., Lemeulle, C., Armanini, M., Simmons, L., Moffet, B., Vandlen, R.A., Simpson LC corrected to Simmons L., et al. (1994). GDNF: a potent survival factor for motoneurons present in peripheral nerve and muscle. Science *266*, 1062–1064.

Hovland, D.N., Boyd, R.B., Butt, M.T., Engelhardt, J.A., Moxness, M.S., Ma, M.H., Emery, M.G., Ernst, N.B., Reed, R.P., Zeller, J.R., et al. (2007). Six-month continuous intraputamenal infusion toxicity study of recombinant methionyl human glial cell line-derived neurotrophic factor (r-metHuGDNF) in rhesus monkeys. Toxicol. Pathol. *35*, 1013–1029.

Hulisz, D. (2018). Amyotrophic lateral sclerosis: disease state overview. Am. J. Manag. Care *24*, S320–S326.

Jomary, C., Darrow, R.M., Wong, P., Organisciak, D.T., and Jones, S.E. (2004). Expression of neurturin, glial cell line-derived neurotrophic factor, and their receptor components in light-induced retinal degeneration. Invest. Ophthalmol. Vis. Sci. *45*, 1240–1246.

Jones, M.K., Lu, B., Saghizadeh, M., and Wang, S. (2016). Gene expression changes in the retina following subretinal injection of human neural progenitor cells into a rodent model for retinal degeneration. Mol. Vis. *22*, 472–490.

Klein, S.M., Behrstock, S., McHugh, J., Hoffmann, K., Wallace, K., Suzuki, M., Aebischer, P., and Svendsen, C.N. (2005). GDNF delivery using human neural progenitor cells in a rat model of ALS. Hum. Gene Ther. *16*, 509–521.

Lepore, A.C., Rauck, B., Dejea, C., Pardo, A.C., Rao, M.S., Rothstein, J.D., and Maragakis, N.J. (2008). Focal transplantation-based astrocyte replacement is neuroprotective in a model of motor neuron disease. Nat. Neurosci. *11*, 1294–1301.

Lepore, A.C., O'Donnell, J., Kim, A.S., Williams, T., Tuteja, A., Rao, M.S., Kelley, L.L., Campanelli, J.T., and Maragakis, N.J. (2011). Human glial-restricted progenitor transplantation into cervical spinal cord of the SOD1 mouse model of ALS. PLoS One *6*, e25968.

Lin, L.F., Doherty, D.H., Lile, J.D., Bektesh, S., and Collins, F. (1993). GDNF: a glial cell line-derived neurotrophic factor for midbrain dopaminergic neurons. Science *260*, 1130–1132.

Liu, Z., Tang, Y., Lü, S., Zhou, J., Du, Z., Duan, C., Li, Z., and Wang, C. (2013). The tumourigenicity of iPS cells and their differentiated derivates. J. Cell Mol. Med. *17*, 782–791.

McBride, J.L., Behrstock, S.P., Chen, E.-Y., Jakel, R.J., Siegel, I., Svendsen, C.N., and Kordower, J.H. (2004). Human neural stem cell transplants improve motor function in a rat model of Huntington's disease. J. Comp. Neurol. *475*, 211–219.

McGill, T.J., Lund, R.D., Douglas, R.M., Wang, S., Lu, B., Silver, B.D., Secretan, M.R., Arthur, J.N., and Prusky, G.T. (2007). Syngeneic Schwann cell transplantation preserves vision in RCS rat without immunosuppression. Invest. Ophthalmol. Vis. Sci. *48*, 1906–1912.

Mendez, I., Sanchez-Pernaute, R., Cooper, O., Viñuela, A., Ferrari, D., Björklund, L., Dagher, A., and Isacson, O. (2005). Cell type analysis of functional fetal dopamine cell suspension transplants in the striatum and substantia nigra of patients with Parkinson's disease. Brain *128*, 1498–1510.

Nutt, J.G., Burchiel, K.J., Comella, C.L., Jankovic, J., Lang, A.E., Laws, E.R., Lozano, A.M., Penn, R.D., Simpson, R.K., Stacy, M., et al. (2003). Randomized, double-blind trial of glial cell line-derived neurotrophic factor (GDNF) in PD. Neurology *60*, 69–73.

Okano, H. (2022a). A combined stem-cell-gene therapy strategy for ALS. Nat. Med. *28*, 1751–1752.

Okano, H. (2022b). Transplantation of neural progenitor cells into the human CNS. Trends Mol. Med. *28*, 897–899.

Ostenfeld, T., Caldwell, M.A., Prowse, K.R., Linskens, M.H., Jauniaux, E., and Svendsen, C.N. (2000). Human neural precursor cells express low levels of telomerase in vitro and show diminishing cell proliferation with extensive axonal outgrowth following transplantation. Exp. Neurol. *164*, 215–226.

Rosati, J., Ferrari, D., Altieri, F., Tardivo, S., Ricciolini, C., Fusilli, C., Zalfa, C., Profico, D.C., Pinos, F., Bernardini, L., et al. (2018). Establishment of stable iPS-derived human neural stem cell lines suitable for cell therapies. Cell Death Dis. *9*, 937.

Sandrock, R.W., Wheatley, W., Levinthal, C., Lawson, J., Hashimoto, B., Rao, M., and Campanelli, J.T. (2010). Isolation, characterization and preclinical development of human glial-restricted progenitor cells for treatment of neurological disorders. Regen. Med. *5*, 381–394.

Sareen, D., McMillan, E., Ebert, A.D., Shelley, B.C., Johnson, J.A., Meisner, L.F., and Svendsen, C.N. (2009). Chromosome 7 and 19 trisomy in cultured human neural progenitor cells. PLoS One *4*, e7630.

Sareen, D., Gowing, G., Sahabian, A., Staggenborg, K., Paradis, R., Avalos, P., Latter, J., Ornelas, L., Garcia, L., and Svendsen, C.N. (2014). Human induced pluripotent stem cells are a novel source of neural progenitor cells (iNPCs) that migrate and integrate in the rodent spinal cord. J. Comp. Neurol. *522*, 2707–2728.

Shelley, B.C., Gowing, G., and Svendsen, C.N. (2014). A cGMP-applicable expansion method for aggregates of human neural stem and progenitor cells derived from pluripotent stem cells or fetal brain tissue. J. Vis. Exp., 51219.

Suzuki, M., McHugh, J., Tork, C., Shelley, B., Klein, S.M., Aebischer, P., and Svendsen, C.N. (2007). GDNF secreting human neural progenitor cells protect dying motor neurons, but not their projection to muscle, in a rat model of familial ALS. PLoS One *2*, e689.

Svendsen, C.N. (2013). Back to the future: how human induced pluripotent stem cells will transform regenerative medicine. Hum. Mol. Genet. *22*, R32–R38.

Svendsen, C.N., Caldwell, M.A., Shen, J., ter Borg, M.G., Rosser, A.E., Tyers, P., Karmiol, S., and Dunnett, S.B. (1997). Long-term survival of human central nervous system progenitor cells transplanted into a rat model of Parkinson's disease. Exp. Neurol. *148*, 135–146.

Svendsen, C.N., ter Borg, M.G., Armstrong, R.J., Rosser, A.E., Chandran, S., Ostenfeld, T., and Caldwell, M.A. (1998). A new method for the rapid and long term growth of human neural precursor cells. J. Neurosci. Methods *85*, 141–152.

Thomsen, G.M., Gowing, G., Latter, J., Chen, M., Vit, J.-P., Staggenborg, K., Avalos, P., Alkaslasi, M., Ferraiuolo, L., Likhite, S., et al. (2014). Delayed disease onset and extended survival in the SOD1G93A rat model of amyotrophic lateral sclerosis after suppression of mutant SOD1 in the motor cortex. J. Neurosci. *34*, 15587–15600.

Thomsen, G.M., Alkaslasi, M., Vit, J.P., Lawless, G., Godoy, M., Gowing, G., Shelest, O., and Svendsen, C.N. (2017). Systemic injection of AAV9-GDNF provides modest functional improvements in the SOD1G93A ALS rat but has adverse side effects. Gene Ther. *24*, 245–252.

Thomsen, G.M., Avalos, P., Ma, A.A., Alkaslasi, M., Cho, N., Wyss, L., Vit, J.-P., Godoy, M., Suezaki, P., Shelest, O., et al. (2018). Transplantation of neural progenitor cells expressing glial cell line-derived neurotrophic factor into the motor cortex as a strategy to treat amyotrophic lateral sclerosis. Stem Cell. *36*, 1122–1131.

Trupp, M., Arenas, E., Fainzilber, M., Nilsson, A.S., Sieber, B.A., Grigoriou, M., Kilkenny, C., Salazar-Grueso, E., Pachnis, V., and Arumäe, U. (1996). Functional receptor for GDNF encoded by the c-ret proto-oncogene. Nature *381*, 785–789.

Tsai, Y., Lu, B., Bakondi, B., Girman, S., Sahabian, A., Sareen, D., Svendsen, C.N., and Wang, S. (2015). Human iPSC-derived neural progenitors preserve vision in an AMD-like model. Stem Cell. *33*, 2537–2549.

Wang, S., Girman, S., Lu, B., Bischoff, N., Holmes, T., Shearer, R., Wright, L.S., Svendsen, C.N., Gamm, D.M., and Lund, R.D. (2008). Long-term vision rescue by human neural progenitors in a rat model of photoreceptor degeneration. Invest. Ophthalmol. Vis. Sci. *49*, 3201–3206.

Wright, L.S., Prowse, K.R., Wallace, K., Linskens, M.H.K., and Svendsen, C.N. (2006). Human progenitor cells isolated from the developing cortex undergo decreased neurogenesis and eventual senescence following expansion in vitro. Exp. Cell Res. *312*, 2107–2120.

Yamanaka, S. (2020). Pluripotent stem cell-based cell therapy-promise and challenges. Cell Stem Cell *27*, 523–531.

Zhang, S., and Cui, W. (2014). Sox2, a key factor in the regulation of pluripotency and neural differentiation. World J. Stem Cells *6*, 305–311.

Zurn, A.D., Baetge, E.E., Hammang, J.P., Tan, S.A., and Aebischer, P. (1994). Glial cell line-derived neurotrophic factor (GDNF), a new neurotrophic factor for motoneurones. Neuroreport *6*, 113–118.

Stem Cell Reports, Volume *18*

Supplemental Information

Human iPSC-derived neural progenitor cells secreting GDNF provide protection in rodent models of ALS and retinal degeneration

Alexander H. Laperle, V. Alexandra Moser, Pablo Avalos, Bin Lu, Amanda Wu, Aaron Fulton, Stephany Ramirez, Veronica J. Garcia, Shaughn Bell, Ritchie Ho, George Lawless, Kristina Roxas, Saba Shahin, Oksana Shelest, Soshana Svendsen, Shaomei Wang, and Clive N. Svendsen

Supplemental Materials

Supplemental Figures

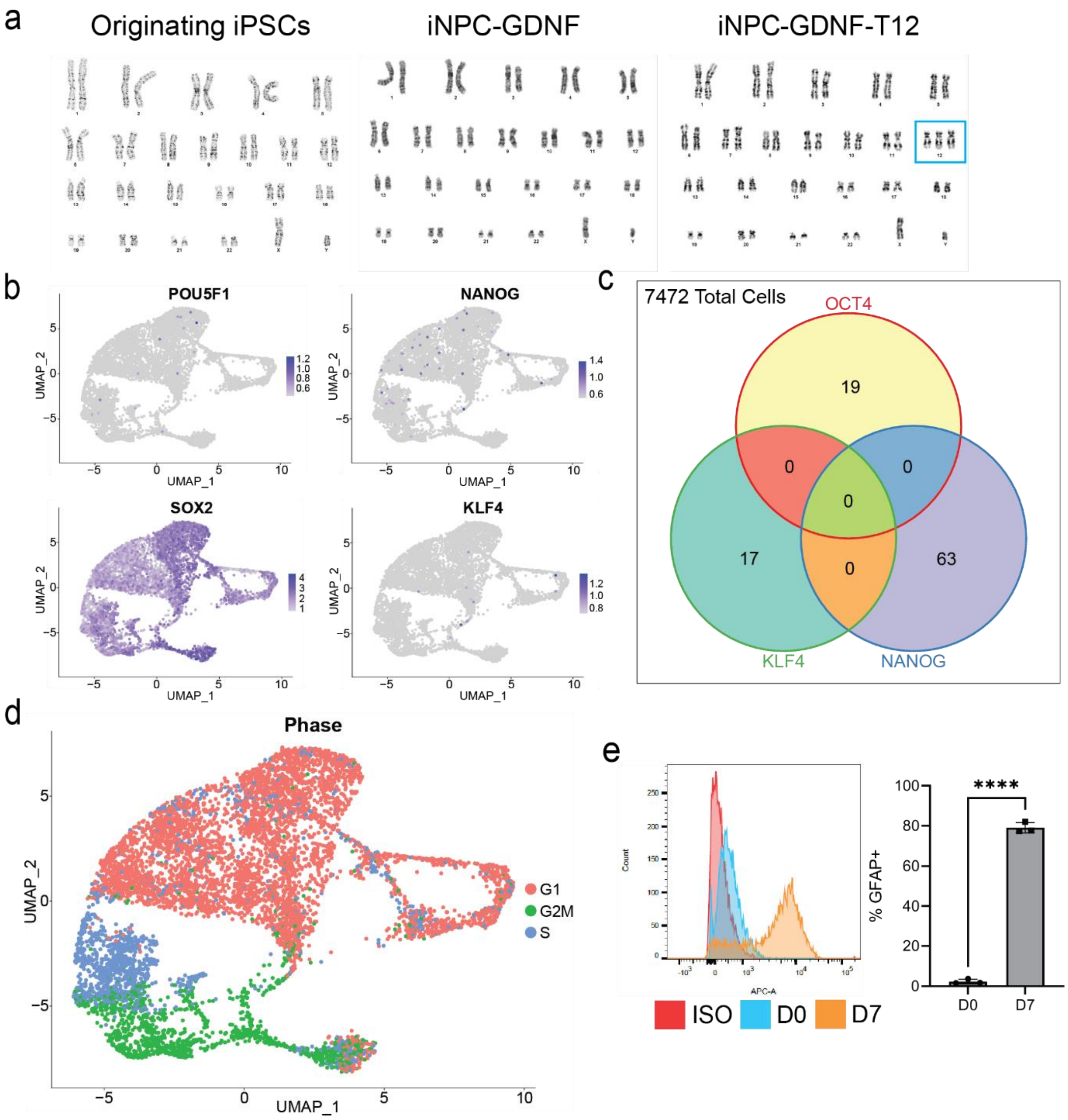

Figure S1: Extended iNPC-GDNF characterization, related to Figure 1: (a) G-band karyotype of originating iPSCs and two iNPC-GDNF batches. iNPC-GDNF-WT retained a normal karyotype while iNPC-GDNF-T12 developed nearly 100% trisomy of chromosome 12 (blue box). (b) Feature plots show expression of pluripotency genes. (c) Venn diagram of cells expressing multiple pluripotency genes. (d) snRNA-seq clustering grouped by cell cycle. (e) Histograms comparing GFAP levels at D0 and D7, with a control of isotype-stained cells (ISO), and quantification of % GFAP+ cells at D0 and D7. **** p<0.0001 via unpaired t test with Welch's correction.

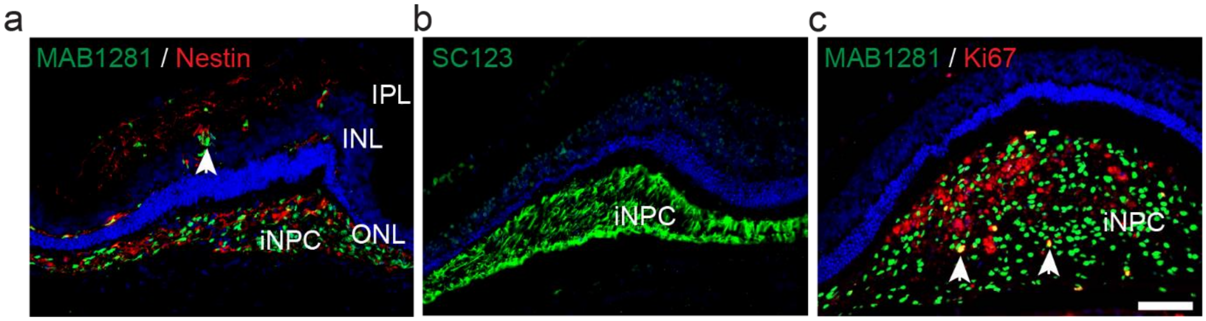

Figure S2: Extended characterization of iNPC-GDNFs in the RCS rat eye, related to Figure 2. Grafted iNPC-GDNFs from D90 RCS rat eyes were positive for MAB1281 and human nestin (a) and SC123 (b). (c) MAB1281 and Ki67 staining shows minimal double-positive cells (arrowheads). Scale bar = 75 μm. INL: Inner nuclear layer, IPL: Inner plexiform layer, ONL: Outer nuclear layer.

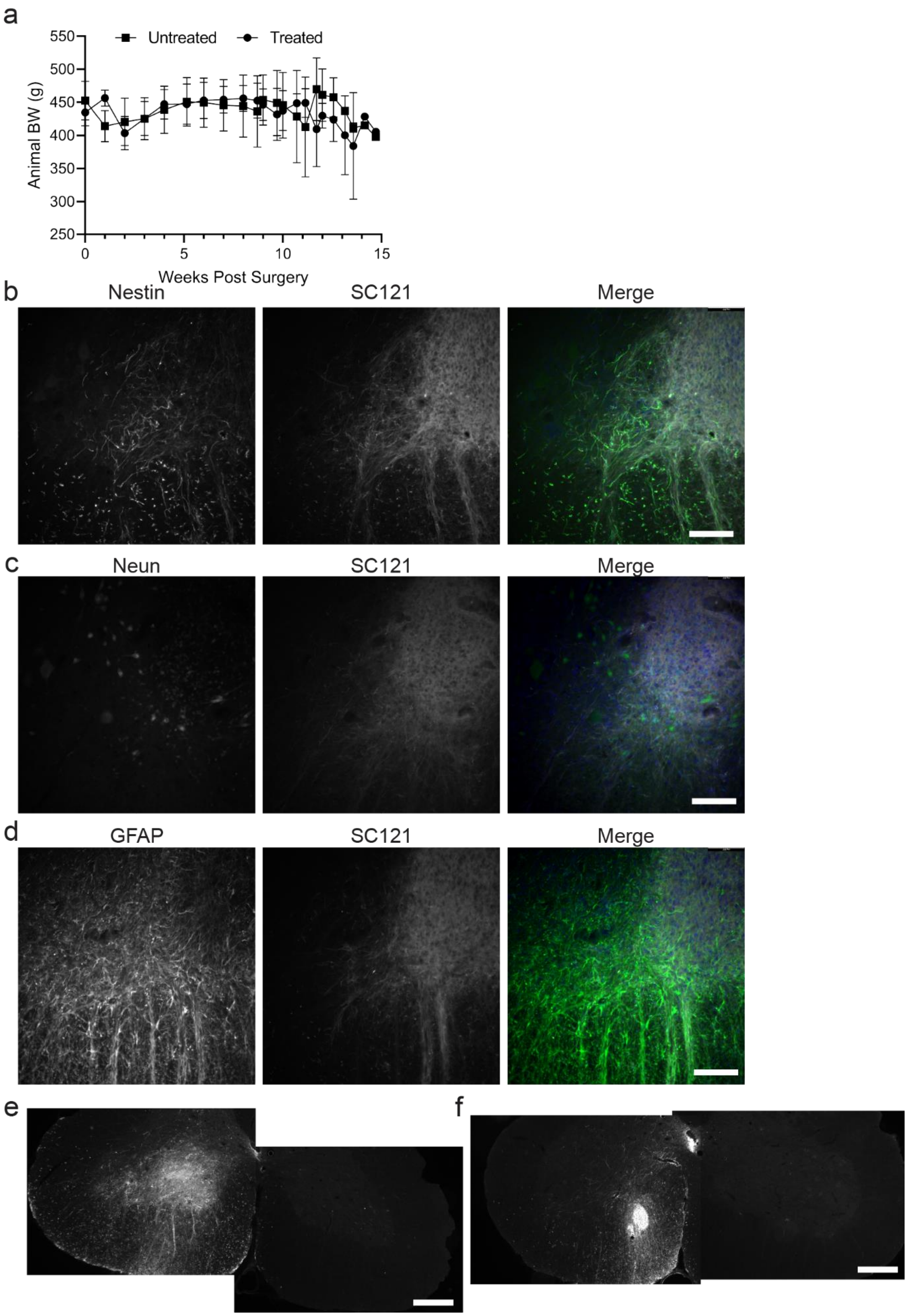
a
Untreated
Treated
Animal BW (g)
550
500
450
400
350
300
250
0
5
10
15
Weeks Post Surgery
b
Nestin
SC121
Merge
c
Neun
SC121
Merge
d
GFAP
SC121
Merge
e
f

Figure S3: Extended characterization of iNPC-GDNFs in the SOD1^{G93A} rat spinal cord, related to Figure 3. (a) Body weight (grams) of all animals after transplantation are similar between treated and untreated rats. Immunofluorescence showing iNPC-GDNFs stained with SC121 along with (b) Nestin, (c) Neun, and (d) GFAP. Immunohistochemistry showing SC121 in the spinal cord of (e) animal #239 (highest treated limb BBB score at onset) and (f) animal #240 (lowest treated hindlimb BBB score at onset). Scale bars = 100 μm (b-d) and 250 μm (e,f).

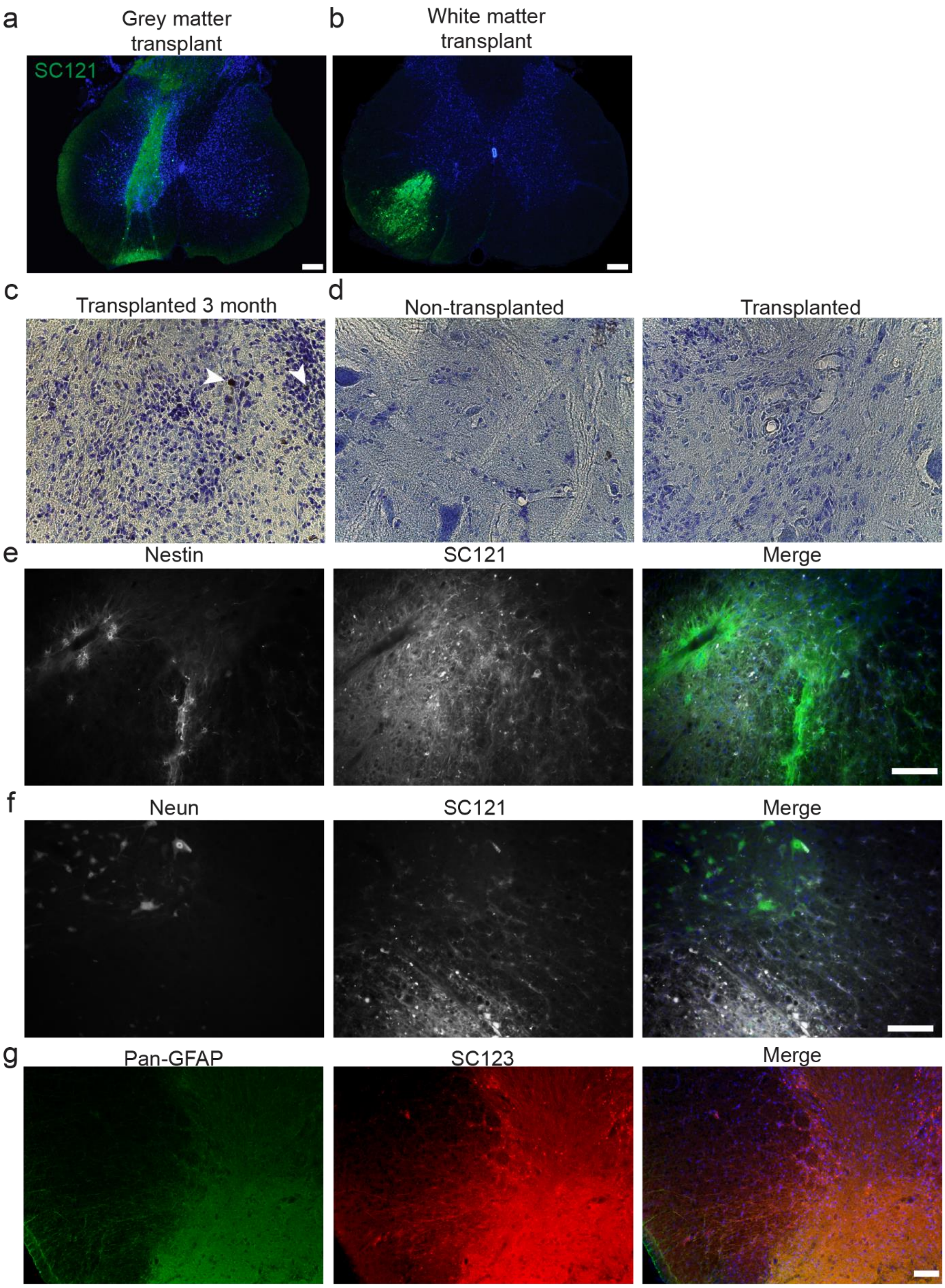
a
Grey matter transplant
SC121
b
White matter transplant
c
Transplanted 3 month
d
Non-transplanted
Transplanted
e
Nestin
SC121
Merge
f
Neun
SC121
Merge
g
Pan-GFAP
SC123
Merge

Figure S4: Extended characterization of long-term iNPC-GDNF grafts in the nude rat spinal cord, related to Figure 4. Grafts at 9 months stained for (a) SC121. Ki67 immunohistochemistry with H&E counterstain of grafts at (b) 3- and (c) 9-months post-transplantation. White arrows indicate examples of positive cells. Immunofluorescence showing iNPC-GDNF stained with SC121 along with (e) Nestin and (f) Neun. (g) pan-GFAP and human-specific GFAP (SC123). Scale bars = 250 µm (a,b) and 100 µm (e-g).

Supplemental Table

Cluster	fNPC-GDNF	iNPC-GDNF
0	95%	5%
1	33%	67%
2	0%	100%
3	36%	64%
4	1%	99%
5	37%	63%
6	52%	48%
7	94%	6%
8	100%	ND
9	53%	47%
10	100%	ND

Table S1: snRNA-seq cluster composition, related to Figure 1. Percent contribution of each cluster by cell source**.**

Supplemental Experimental Procedures

Materials

Reagent	Manufacturer	Catalogue #
E8 media	Produced in house	N/A
Versene	Thermo Fisher Scientific	15040066
Accutase	Sigma	SCR005
PS TC plates	Fisher	08-772-1B
Y27632	Stemgent	04-0012-02
DMEM/F12	Thermo Fisher Scientific	11320082
Neurobasal	Thermo Fisher Scientific	21103049
N2	Thermo Fisher Scientific	17502048
B27-vitamin A	Thermo Fisher Scientific	12587010
MEM Non-Essential Amino Acids Solution	Thermo Fisher Scientific	11140050
L-glutamine	Thermo Fisher Scientific	25030081
Human insulin	Thermo Fisher Scientific	12585014
LDN-193189	Stemgent	04-0074-02
SB431542	Cayman Chemicals	13031
Stemline media	Sigma	S3194
Human EGF	Peprotech	AF-100-15
Human FGF2	Peprotech	100-18B
Human LIF	Sigma	LIF1010
Heparin	Sigma	H3149
Poly-HEMA	Thermo Fisher Scientific	P3932
PS TC Flasks	Thermo Fisher Scientific	29185-308
TrypLE	Thermo Fisher Scientific	12604013
cGMP-grade lenti-SIN-WP-mPGK-GDNF	Indiana University	N/A
Cell freezing medium	Sigma	C6295
poly-l-ornithine (PLO)	Sigma	P4638
Matrigel	Corning	354230
Paraformaldehyde (PFA)	Fisher	50-980-495
Phosphate buffered saline (PBS)	Fisher	MT21031CV
Triton X-100	Fisher	AAA16046AP
Tris buffered saline (TBS)	Bio-Rad	1706435
Normal horse serum	Vector	S-2000-20
Normal donkey serum	Sigma	566460
Bovine serum albumin	Sigma	A9418
Vectashield mounting medium	Vector	H-1000-10
Hematoxylin	Sigma	HHS3201L
Lithium carbonate	Rowley Biochemicals	K-680-1
0.1% ammonia water	Electron Microscopy Sciences	26698-02
Eosin	Sigma	HT110332
Permanent mounting media	Thermo Fisher Scientific	4112

Antibodies

Application	Antibody	Manufacturer	Catalogue #	Dilution
ICC	GFAP	Dako	Z0334	1:2000
ICC	S100β	Sigma	S2532-.2ML	1:1000
ICC, IHC	Alexa Fluor 488	Invitrogen	A21206	1:500
ICC, IHC	Alexa Fluor 594	Invitrogen	A21203	1:500
Flow	Alexa Fluor 647	Invitrogen	A32733	1:500
ICC, IHC	49,69-diamidino-2-phenylindole (DAPI)	Thermo Fisher Scientific	D1306	300nM
IHC	Cone Arrestin	Millipore	AB15282	1:10,000
IHC	Synaptophysin	Millipore	573822	1:2,000
IHC	Human Nuclei	Millipore	MAB1281	1:300
IHC	Human Nestin (eye)	Millipore	ABD69	1:2,000
IHC	Human Nestin (spinal cord)	Millipore	ABD69	1:500
IHC	GFAP (eye)	Millipore	SAB2107063	1:1,000
IHC	GFAP (spinal cord)	Dako	Z0334	1:2000
IHC	Ki67 (eye)	Millipore	AB9260	1:500
IHC	Ki67 (spinal cord)	Thermo Fisher Scientific	RM-9106-S	1:200
IHC	SC123	Takara Bio	Y40420	1:500
IHC	SC121 (spinal cord)	Takara Bio	Y40410	1:50
IHC	GDNF (spinal cord)	R&D Systems	BAF212	1:50
IHC	ChAT	R&D Systems	AF3447	1:100
IHC	IBA1	Novus Biologicals	NB100-1028	1:300
IHC	Neun	Cell Signaling	24307	1:250
IHC	Biotinylated Secondaries	Vector	Vector, BA-9500 or BA-1100	1:200
IHC	Avidin-Biotin Complex	Vector	PK-4000	N/A
IHC	DAB kit	Vector	SK-4100	N/A
IHC	Alexa Fluor 488 (anti Goat)	Invitrogen	A11055	1:500
Flow	GFAP	Dako	Z0334	1:250
Flow	Rabbit isotype control	Cell Signaling	3900S	1:250

Cell culture media formulations

Differentiation Media	Reagent
50%	DMEM/F12
50%	Neurobasal
1%	N2
2%	B27-vitamin A
1%	MEM Non-Essential Amino Acids Solution
2mM	L-glutamine
5µg/mL	human insulin
1µM	LDN-193189
2µM	SB431542

SEFL Media	Reagent
Full Volume	Stemline media
100ng/mL	human EGF
100ng/mL	human FGF2
10ng/mL	human LIF
5µg/mL	Heparin

Single Nuclei RNA Sequencing

Nuclei isolation: Nuclei isolation was based on a modified published protocol (Corces et al., 2017). All steps were performed on ice. Frozen cell pellets were thawed on ice, resuspended in cold homogenization buffer (5mM $MgCl_2$, 3mM $Mg(Ac)_2$, 10mM Tris pH7.8, 0.017 mM PMSF, 0.17 mM β-mercaptoethanol (β-Me), 320 mM Sucrose, 0.1 mM EDTA, 0.1% NP40, RNAse inhibitors, and Protease inhibitors), and transferred to a glass dounce. Cells were manually lysed. Homogenate was filtered using 70 um Flowmi pipette tip filters and gently mixed 1:1 with a 50% Iodixanol solution (5mM $MgCl_2$, 3mM $Mg(Ac)_2$, 10mM Tris pH7.8, 0.017 mM PMSF, 0.17 mM β-Me, RNAse inhibitors, Protease inhibitors, and 50% Iodixanol). A centrifugation gradient was set up using 600 μl 40% Iodixanol solution (5mM $MgCl_2$, 3mM $Mg(Ac)_2$, 10mM Tris pH7.8, 0.017 mM PMSF, 0.17 mM β-Me, RNAse inhibitors, Protease inhibitors, 160 mM Sucrose, 0.2 μg/μl OrangeG, and 40% Iodixanol), 600 μl 29% Iodixanol solution (5mM $MgCl_2$, 3mM $Mg(Ac)_2$, 10mM Tris pH7.8, 0.017 mM PMSF, 0.17 mM β-Me RNAse inhibitors, Protease inhibitors, 160 mM Sucrose, and 29% Iodixanol) and 800 μl of the 50% Iodixanol + Sample mixture (25% Iodixanol final concentration). This was centrifuged at 3000 x g for 1 hour with breaks disengaged. Any debris on the surface was removed, and 200 μl of the thin cloudy layer containing the nuclei was extracted, mixed with 1.8 ml PBS + 1% BSA solution, and centrifuged at 500 x g for 10 minutes with breaks engaged. 1.8 ml supernatant was removed and the remaining 200 μl plus nuclei pellet was resuspended in 1.8 ml PBS + 1% BSA solution. The nuclei suspension was counted via hemocytometer and checked for nucleus quality. A portion of this suspension was diluted to the correct nuclei concentration for the 10x Chromium NextGEM 3' protocol with a target of ~4,000 nuclei per sample.

Single nuclei library preparation and sequencing: The standard 10x protocol was used per the "Chromium NextGEM Single Cell 3' Reagent Kits v3.1 User Guide, Rev D" (single index). Briefly, nuclei were resuspended in the master mix and loaded together with partitioning oil and gel beads into the chip to generate the gel bead-in-emulsion (GEM). The poly-A RNA from the nucleus lysate contained in every single GEM was retrotranscripted to cDNA, which contains an Ilumina R1 primer sequence, Unique Molecular Identifier (UMI) and the 10x Barcode. The pooled, barcoded cDNA was then cleaned up with Silane DynaBeads, amplified by PCR, and the appropriately sized fragments were selected with SPRIselect reagent for subsequent library construction. The amplified cDNA and sequencing libraries were quality checked using an Agilent 2100 BioAnalyzer (Agilent Technologies, Santa Cruz CA) using a High Sensitivity DNA chip. Barcoded sequencing libraries were quantified by qPCR on a QuantStudio12k Flex (Thermo Fisher Scientific, Waltham, MA) system using the Collibri Library Quantification Kit (Thermo Fisher Scientific). The uniquely indexed libraries were pooled at equal ratio and sequenced on a NovaSeq 6000 (Illumina, San Diego, CA), with a sequencing depth of ~50,000 reads/cell. Raw sequencing data were demultiplexed and converted to FASTQ format using bcl2fastq v2.20.

Single nuclei data analysis: Demultiplexed fastq files were run via CellRanger v6.1.2 using the "cellranger count" command with the "--include-introns" option using the precompiled CellRanger human reference sequence “v2020-A” provided on the 10x Genomics website. CellRanger output “filtered_feature_bc_matrix files” (barcodes.tsv.gz, features.tsv.gz & matrix.mtx.gz) were loaded into R (v4.2.1, https://www.R-project.org/) via Rstudio (v2022.07.1, Build 554) and combined into one matrix file per sample with genes as rows (with both gene symbol and Ensmbl ID) and cells (nuclei) as columns. Each matrix file was then processed by summing all Ensmbl IDs with the same gene symbol to the gene symbol level via standard R matrix processing functions. The gene-summed matrices were loaded into the Seurat R package (v4.1.1) keeping all genes that

were expressed in more than one cell (Hao et al., 2021). All samples were merged into a single Seurat object (7,959 nuclei) and filtered by removing all cells with counts, genes, mitochondrial gene percentage, and ribosomal gene percentage outside of three standard deviations from the mean (7,472 nuclei). The samples were then split and normalized, and variable features were identified. Integration features were then selected and used to scale the data and run principal component analysis (PCA). Integration anchors were identified via the Seurat function "FindIntegrationAnchors()", and these anchors were used to integrate the samples into a single Seurat object. The integrated object was scaled again to account for the scale of the whole data set. PCA was run on the integrated object using 50 principal components (PCs). The number of "meaningful PCs" used in subsequent analysis was determined by comparing the actual contributed variance of each PC versus the hypothetical situation where each PC would contribute equally to the variance. 13 PCs contributed more variance than the calculated hypothetical equal-variance level and were considered "meaningful". These 13 PCs were used in the subsequent analysis. Next, the nuclei were clustered using the Seurat functions "RunUMAP()", "FindNeighbors()", and "FindClusters()". A resolution factor of 0.5 identified 10 distinct clusters. For generation of UMAP plots, the RNA slot in the integrated Seurat object was processed with the Seurat functions "NormalizeData()", "FindVariableFeatures()", and "ScaleData()" using all genes present in the data set for the "features" option of "ScaleData()". The Venn diagram was generated using the "Venn()" function in the Vennerable R package (https://github.com/js229/Vennerable).

Flow cytometry: Cells from 3 cryopreserved vials of iNPC-GDNFs were split at thaw with half fixed immediately in 4% paraformaldehyde (PFA) (D0) and half plated for 7 days (D7) for differentiation as a monolayer culture. Monolayer grown cells were washed once in phosphate buffered saline (PBS) then 1mL Accutase was added to each well and incubated for 5 mins at 37°C. Singularized cells were collected and pelleted by centrifugation (1500 RPM for 3 mins). Cells were gently resuspended in 4% PFA in PBS and allowed to fix for 10-15 mins at room temperature. All fixed single cells were permeabilized using 1% Triton X-100 and stained using primary antibodies against GFAP or an isotype control (1:250) overnight at 4 °C. Samples were washed 3 times in PBS with 0.1% TritonX-100 and 5% NDS. Secondary antibodies (Alexa Fluor 647) were used at 1:500 at room temperature for one hour. Stained samples were quantified on an LSR Fortessa cytometer using BD FACSDiva software and analyzed using FloJo software.

Animals: Given our extensive knowledge that neural progenitor cells alone are less effective than cells producing GDNF, and to uphold the 3Rs to "replace, reduce and refine" animal use (Hubrecht and Carter, 2019), studies did not use iNPC alone rat cohorts. Animals were housed and maintained at the Cedars-Sinai Department of Comparative Medicine vivarium. All animal studies were approved and supervised by the Institutional Animal Care and Use Committee and all animals were treated in accordance with the ARVO Statement for the Use of Animals in Ophthalmic and Vision Research.

Visual function testing: All rats were tested by optokinetic response (OKR) and electroretinography (ERG) at approximately P60 and P90, according to our published protocols (Gamm et al,. 2007; Wang et al., 2008). Animals were tested for spatial visual acuity using an optometry testing apparatus. This device consists of a rotating cylinder covered with a vertical sine wave grating presented in virtual three-dimensional (3-D) space on four computer monitors arranged in a square. Unrestrained rats were placed on a platform in the center of the square, where they tracked the grating with reflexive head movements. The spatial frequency of the grating was clamped at the viewing position by repeatedly re-centering the 'cylinder' on the head of the test subject. Acuity was quantified by increasing the spatial frequency of the grating using

a psychophysics staircase progression until the optokinetic reflex was lost, thereby obtaining a maximum threshold. Dark adapted full field ERG responses were recorded following our published protocol (Tsai et al., 2015). Special care was taken to maintain electrode placement in a consistent position in all animals. Averages of 3–5 traces (set 2 minutes apart to ensure recovery of rod responsiveness) were obtained.

Histology and Immunohistochemistry

RCS model: Eyes from the RCS rats were removed and post-fixed in 4% PFA for 1-2 hour, then placed in 10%, 20% sucrose for 1 hour, and placed in 30% sucrose at 4 °C overnight. The corneas and lenses were removed, and the eyes were embedded in optimal cutting temperature compound (OCT). Sectioning was performed on a cryostat and retinal sections were collected in 5 series according to our previous protocol (Tsai et al., 2015). One of every five slides was stained with cresyl violet (CV) and the remaining slides were stored at −80 °C for antibody staining. CV-stained images were acquired with a regular bright field microscope and ProgRes capture system (Jenoptik, Jupiter, FL). Retinal sections were immunohistochemically stained according to our published protocol (Tsai et al., 2015).

Quantification of outer nuclear layer: ONL thickness and donor cell distribution were measured on cresyl violet-stained sections (6 retinas, 6 sections/retina). Retinal montage sections were prepared for measuring the length of the preserved ONL as compared to the whole retinal length. Measurements were made using Java-based image processing software (ImageJ; National Institutes of Health, Bethesda, MD).

$SOD1^{G93A}$ and nude rat models: Spinal cords were removed, post-fixed in 4% PFA for 24 h, and transferred to 30% sucrose until the tissue sank. The lumbar region was then sectioned at 35 μm sections using a microtome. Sections were washed 3 times in PBS for 5 min, quenched in 3% hydrogen peroxide/3% methanol for 10 min, then washed 3 times in 0.5% Triton X-100 in 1X Tris buffered saline (TBS). Sections were then blocked in 5% Normal Horse Serum and 2% Bovine Serum Albumin for 60 mins at room temperature, followed by overnight incubation at 4ºC in primary antibodies. Sections were washed with 0.2% Triton X-100 in 1X TBS 3 times for 10 min, then placed into the appropriate biotinylated or fluorescent secondary antibodies for 1.5 to 2 hours at room temperature. Sections with immunofluorescent staining were washed 3 times for 5 min in 1X TBS, incubated with DAPI for 2 minutes, and washed again with 1X TBS 3 times for 5 min each before being mounted onto slides and coverslipped using Vectashield mounting medium. Sections incubated with biotinylated secondary antibodies were placed in Avidin-Biotin Complex for 1 hour at room temperature and then washed 3 times for 10 min each in 0.2% Triton X-100 in 1X TBS. A DAB kit was used for chromogenic detection with 3,3′-Diaminobenzidine. Sections were then washed twice for 5 min each in 1X TBS, mounted onto slides, dehydrated, and coverslipped with permanent mounting media.

Supplemental References

Corces, M.R., Trevino, A.E., Hamilton, E.G., Greenside, P.G., Sinnott-Armstrong, N.A., Vesuna, S., Satpathy, A.T., Rubin, A.J., Montine, K.S., Wu, B., et al. (2017). An improved ATAC-seq protocol reduces background and enables interrogation of frozen tissues. Nat. Methods *14*, 959–962.

Hao Y., Hao S., Andersen-Nissen E., Mauck W.M. 3rd, Zheng S., Butler A., Lee M.J., Wilk A.J., Darby C., Zager M., et al. (2021) Integrated analysis of multimodal single-cell data. Cell. *184*, 3573–3587.e29.

Hubrecht, R.C., and Carter, E. (2019). The 3rs and humane experimental technique: implementing change. Animals (Basel) *9*.
